# Density-Dependent Prophylaxis in Freshwater Snails Driven by Oxylipin Chemical Cues

**DOI:** 10.3389/fimmu.2022.826500

**Published:** 2022-01-31

**Authors:** Olwyn C. Friesen, Chen-Hua Li, Ellen M. E. Sykes, Jake M. Stout, Harold M. Aukema, Ayush Kumar, Jillian T. Detwiler

**Affiliations:** ^1^ Department of Biological Sciences, University of Manitoba, Winnipeg, MB, Canada; ^2^ Faculty of Veterinary Medicine, University of Calgary, Calgary, AB, Canada; ^3^ Department of Microbiology, University of Manitoba, Winnipeg, MB, Canada; ^4^ Department of Food and Human Nutritional Sciences, University of Manitoba, Winnipeg, MB, Canada; ^5^ Canadian Centre for Agri-Food Research in Health and Medicine, St. Boniface Hospital Research Centre, Winnipeg, MB, Canada

**Keywords:** density-dependent prophylaxis, oxylipins, trematode, snails, immune response

## Abstract

While animal aggregations can benefit the fitness of group members, the behaviour may also lead to higher risks of parasite infection as group density increases. Some animals are known to moderate their investment in immunity relative to the risk of infection. These animals exhibit density-dependent prophylaxis (DDP) by increasing their immune investment as group density increases. Despite being documented in many taxa, the mechanisms of DDP remain largely unexplored. Snails are known to aggregate and experience large fluctuations in density and serve as required hosts for many parasites. Further, they are known to use chemical cues to aggregate. To test whether freshwater snails exhibit DDP and investigate the role that chemical signaling compounds may play in triggering this phenomenon, we performed four experiments on the freshwater snail *Stagnicola elodes*, which is a common host for many trematode parasite species. First, we tested if DDP occurred in snails in laboratory-controlled conditions (control vs snail-conditioned water) and whether differences in exposure to chemical cues affected immune function. Second, we used gas chromatography to characterize fatty acids expressed in snail-conditioned water to determine if precursors for particular signaling molecules, such as oxylipins, were being produced by snails. Third, we characterized the oxylipins released by infected and uninfected field-collected snails, to better understand how differences in oxylipin cocktails may play a role in inducing DDP. Finally, we tested the immune response of snails exposed to four oxylipins to test the ability of specific oxylipins to affect DDP. We found that snails exposed to water with higher densities of snails and raised in snail-conditioned water had higher counts of haemocytes. Additionally, lipid analysis demonstrated that fatty acid molecules that are also precursors for oxylipins were present in snail-conditioned water. Trematode-infected snails emitted 50 oxylipins in higher amounts, with 24 of these oxylipins only detected in this group. Finally, oxylipins that were higher in infected snails induced naïve snails to increase their immune responses compared to sham-exposed snails. Our results provide evidence that snails exhibit DDP, and the changes in oxylipins emitted by infected hosts may be one of the molecular mechanisms driving this phenomenon.

## 1 Introduction

The aggregation of animals is evolutionarily beneficial, increasing reproductive success and survival, yet may come with higher risks of parasite or pathogen infection (1, 2). As parasites frequently exhibit density-dependent transmission, the *per capita* risk of infection for an individual host generally increases as host population density increases (3). Individuals that experience fluctuating or high population densities would benefit from investing in immune defence, yet maintaining their defences in the absence of infection can be costly (4, 5). Therefore, natural selection should favour prophylactic investment in immunity that responds to density-dependent parasite transmission rates and the cost of maintaining parasite resistance (6, 7). If species can moderate their level of investment to match that of the perceived risk of infection, then their immune investment should increase with population densities, known as the density-dependent prophylaxis (DDP) phenomenon. Most research focusing on DDP has found supporting evidence in arthropod taxa, particularly in insect species (6, 8, 9). However, as few non-arthropod invertebrate taxa have been investigated for DDP, the evolution of this phenomenon and its underlying molecular mechanisms are not well understood (2, 10).

Many invertebrate taxa, including snails, are known to aggregate and experience fluctuations in population densities from very low to very high (11, 12) and may use DDP to respond to corresponding changes in their infection risk. Snail aggregation has been demonstrated in response to both abiotic and biotic factors, such as dissolved oxygen, water pH, macrophytes and conspecifics (13–16). Such aggregations increase the likelihood that helminth parasites could be transmitted from snail to snail. Common parasites of snails include trematodes, with almost all of the approximately 18,000 described species using molluscs as first intermediate hosts (17). Within the first intermediate host, progenitor larval stages (rediae/sporocysts) produce trematode cercariae that subsequently emerge from the host snail to seek out the next host in the life cycle (second intermediate host). Although calculations of cercarial output are not known for many trematode species, there are some estimates for species that use snails as first and second intermediate hosts. Studies on *Echinostoma trivolvis* and *Echinostoma caproni* found that first intermediate freshwater snail hosts had a daily cercarial output ranging from 431-1981 (18, 19). Therefore, even a low number of infected first intermediate snail hosts could increase the risk of echinostome infection to other snails in close proximity. Freshwater snails can also serve as the second intermediate hosts for many trematode parasites that are found in different and diverse families such as Echinostomatidae Looss, 1899, Psilostomidae Looss, 1900, Strigeidae Railliet, 1919 and Cyathocotylidae Mühling, 1898 (20–22). Among echinostome parasites, there are at least 34 species using freshwater snails as second intermediate hosts (22). Thus, in response to the ubiquitous and frequent risk of trematode parasitism, snail hosts as well as other invertebrate second intermediate hosts may have evolved and maintained DDP.

The immune response of snails, coordinated by haemocytes and humoral factors, is key to its defence against infection (23, 24). Snail haemocytes, part of a snail’s cellular immunity, are indicators of immune response as they phagocytize and encapsulate foreign materials (including cercariae) and produce cytotoxic reactive oxygen species (23, 25–27). In addition, phenoloxidase (PO) systems are considered to be an important component of the humoral response in many invertebrates, with these enzymes playing key roles in wound healing and defence against eukaryotic pathogens (28, 29). These enzymes serve as the last component of the “proPO activating system” reaction cascade (29–31). Antimicrobial proteins are also key to snail immune response and are used to resist microbial infections (32, 33). There is evidence that immune function is costly because it can lead to a reduction in snail growth or reproduction (34, 35). Given these costs, snails experiencing a reduced risk of parasitism (i.e., lower group density) may reduce their constitutive defences, instead relying on an inducible defence to invest more energy in other life history traits (36).

Although laboratory-based studies suggest DDP in invertebrates, one challenge to understanding the mechanisms behind this phenomenon is to determine how animals recognize that they are in a group and therefore at an increased risk of parasitism. Within aquatic communities, species are known to obtain crucial information about their surroundings, such as the presence of other species and conspecifics, through chemical cues that create signaling webs (37–39). Gradual shifts in the diversity and concentration of cues within the water can be used by species to gain information about their environment, and mediate interactions such as predator avoidance and parasite transmission (38–42). Many freshwater snails can attract conspecific or heterospecific snails through the release of chemical cues into the water (43, 44).

Species-specific chemical cocktails released by aquatic organisms can include fatty acids, amino acids, nucleotides, and other volatile organic compounds (37, 42, 45). Lipid analysis suggests that aquatic invertebrates accumulate higher levels of C20 polyunsaturated fatty acids compared to terrestrial insects, suggesting their potential importance in aquatic chemical communication (46). Lipids have also been identified from snail-conditioned water (47–49). Some of these lipids are oxygenated metabolites of polyunsaturated fatty acids, known as oxylipins (50, 51). Oxylipins influence many functional aspects of an organism’s biology. In molluscs, oxylipins affect the neuro- and reproductive physiology and their interactions with parasites (42, 46, 50, 52–55). Intriguingly, oxylipins are not just produced by free-living organisms. Some parasites, including the human trematode *Schistosoma mansoni*, synthesize oxylipins that play a role in parasite development, penetration of the host, and modulating host defences (46, 56–58). However, considering the diversity of invertebrate hosts and their parasites, the function and ecological role of oxylipins is largely unknown (55, 57, 58). To our knowledge, the function of oxylipins in snail host aggregation behaviour and DDP has yet to be determined.

To test for evidence of DDP and the potential role of oxylipins in the phenomenon, we focused on the freshwater snail, *Stagnicola elodes* Say 1821 (also known as *Lymnaea elodes* and *Ladislavella elodes)*, which commonly serve as snail intermediate hosts for many trematode parasites (59). In our experiments, we used *Echinoparyphium* spp. parasites, which are distributed throughout wetlands in North America (60, 61). Freshwater snails serve as first intermediate hosts in which the rediae stage produces free-swimming parasite larvae (cercariae) that upon maturity emerge into the water. Cercariae then seek out a second intermediate host which can be a conspecific or heterospecific snail. Upon encountering a potential second intermediate host, cercariae penetrate and migrate primarily to the pericardial sac and kidney areas, and then encyst as metacercariae (62).

To better understand whether freshwater snails exhibited DDP and investigate the potential role chemical signaling compounds may play in triggering this phenomenon, we performed four sets of experiments. For the first experiment, we had two objectives. First, we tested to see if snails elicited prophylaxis in response to increased snail density. Second, we tested the role of chemical communication in DDP of snails. We hypothesized that if snails exhibited DDP using chemical communication, then snails raised in snail-conditioned water would produce more haemocytes than snails raised in control water. For our second experiment, we investigated if there was a difference in the fatty acid chemical cues emitted in the water with and without snails. Further, we confirmed whether these fatty acids were progenitor molecules of oxylipins that may be a potential mechanism for DDP. For our third experiment, we characterized the diversity and amount of oxylipins released by infected and uninfected field-collected *S. elodes* snails. Using high-performance liquid chromatography/tandem mass spectrometry (HPLC/MS/MS), we identified and measured the oxylipins present in snail-conditioned water. If oxylipin cocktails from infected snails differ in diversity and amounts of oxylipins compared to uninfected snails, then the production of these oxylipins may play a role in altering transmission success and inducing a DDP response in snails. Finally, for our fourth experiment, we tested the immune response of *S. elodes* snails to four candidate oxylipins to determine the role of oxylipin signaling molecules in DDP. Snails were either sham-exposed (controls) or exposed to an oxylipin and their immune function was assessed at three different times post-exposure (1-2hrs, 4-6hrs, and 8-10hrs) to test for the role of these oxylipins in triggering the snail immune system. We chose four oxylipins based on the results of experiment 3, with three oxylipins found in higher amounts in infected snails and one only produced by uninfected snails. We hypothesized that if any of the candidate oxylipins are involved in triggering prophylaxis in snails, then we would see an increased immune response in snails exposed to the oxylipin compared to sham-exposed snails. We also hypothesized that if the immune response is costly, then snails will only elicit an immune response at a particular time point post-exposure rather than throughout the entire exposure period.

## 2 Materials and Methods

### 2.1 Study System

*Stagnicola elodes* is a freshwater snail that occurs in several different types of wetlands throughout the USA and Canada (63, 64). In addition, these snails are required hosts for several trematode parasites (65). Snails used in this study were collected from an ephemeral wetland in Libau, Manitoba (50°16’4.80” N -96°43’5.39” W). For experiments 1, 2, and 4, we used offspring of field-collected individuals to create snail colonies to ensure that snails were trematode-free and to control for genetic variation in immunocompetence among the snails (32). All snails were kept at 22.8°C under a 12hr light:12hr dark regime and were fed lettuce *ad libitum* and water-changed biweekly. To assess the oxylipins emitted by infected and uninfected snails, we collected field snails in June-August 2017 and 2018. Field-collected snails were maintained in non-chlorinated water (called well water from hereon) in 10 L plastic containers and fed lettuce *ad libitum*.

### 2.2 Experiment 1: Do Snails Exhibit Density-Dependent Prophylaxis?

Hatchling snails were lab-raised individually in 100mL containers and used to test for density-dependent prophylaxis within a 12hr period and over an extended period (3 weeks). For both trials, hatchling *S. elodes* were lab-raised individually in 100mL containers and randomly assigned to treatments. Snails were reproductively mature at the time of the experiments, and reproductive output was monitored throughout each experiment, with egg cases and eggs produced by snails counted.

To determine if DDP occurred over a 12hr period, we isolated snail eggs from 30 individuals from the lab colony. Once the eggs hatched, we raised single *S. elodes* snails in individual jars to prevent them from being exposed to oxylipins from other snails. Water in the jars was changed every 3 days and the snails were fed lettuce every 3 days. We raised 250 hatchling snails individually and randomly assigned them into treatments. All snails were raised in well water provided by the Animal Holding Facility at the University of Manitoba. The snails were allowed to grow to >14mm to allow for sufficient haemolymph collection from each snail and all snails were similar in shell length (mean shell length and SE: 17.04 ± 0.14mm). We first randomly chose 10 snails for the pre-trial group, to establish a baseline immune function. Snails were exposed to either low-density snail-conditioned water, or high-density snail-conditioned water. Snail-conditioned water of low and high densities was created by placing snails (>10mm in shell length) in tanks filled with well water to create two different densities of snails – 14.2 snail/L and 43 snails/L. Before exposing snails to the snail-conditioned water, it was filtered through 0.2µm nylon membranes (Omicron 170047R, 47mm) to remove faeces, bacteria, and other debris. By filtering the water, we ensured any immune response was due to exposure to chemical cues (signaling molecules), and not additional exposure to bacteria.

Snail exposures occurred in clean 100mL glass jars to reduce chances that bacteria or chemicals could affect immune responses (bleached, rinsed with ethanol, and autoclaved). Twenty-four hours prior to exposures, each glass jar was filled with artificial spring water (ASW), see recipe by Ulmer (1970) (66), and 1 snail was transferred to each jar to acclimate to new conditions. The exposures consisted of placing each snail in another clean jar filled with either ASW (pre-trial), low- or high-density snail-conditioned water and left for 1-2hrs, 4-6hrs, or 8-10hrs. Each of these exposure periods could be similar to the amount of time in a day in which a snail is in close proximity to another infected snail or time spent in a group with other infected individuals and cercariae in nature. These exposure periods also occur within the period of time it takes a cercariae to infect a snail, from penetration, to migration, and encystment within its new host (67). At the completion of the exposure, we estimated snail immune defences in response to oxylipin exposure by measuring several immune parameters from haemolymph using methods adapted from Seppälä and Jokela (2010) and Le Clec’h et al. (2016) (29, 32). Snail haemolymph was sampled with autoclaved glass pipettes at each time point and subsampled for haemocyte counts (20µL) and phenoloxidase assays (10µL). Snail shell length was also measured to the nearest 0.01mm. Haemocytes were counted immediately, using ~40µl of 0.4% trypan blue in sterile snail saline (1M•CaCl2, 1M•NaCl, 1M•NaOH, 1M•HEPES, 1M•KCl) to distinguish between live and dead haemocytes on Neubauer hemocytometers (Blau Brand, Wertheim, Germany). Haemolymph subsamples for phenoloxidase assays were mixed with sterilized 100µL phosphate buffered saline (PBS, pH 7.4) and snap frozen with liquid nitrogen. Samples were kept frozen at -80°C until phenoloxidase assays were completed.

For the second trial examining DDP in snails over weeks, 60 hatchling *S. elodes* were randomly assigned to two treatments. Control treatments consisted of 30 hatchlings raised individually in well water (control). The experimental treatment was 30 snails raised individually in water taken from a 5L tank filled with 60 *S. elodes* (>10mm in length). This tank was established three weeks prior to the start of the experiment and maintained throughout the experiment to ensure that snail-conditioned water was available. Each week, approximately 800 mL of well water was added to the tank due to use in the experiments and evaporation. The water in the control and experimental containers was changed biweekly. After eight weeks, haemocytes were counted, as described above, and shell length was recorded weekly for three weeks. In addition, 1L of snail-conditioned water and control water was frozen at -20°C after each haemocyte count for lipid analysis (experiment 2). Snail-conditioned water samples were collected from the upper layer of the 5L tank with 60 snails to avoid faeces.

#### 2.2.1 Phenoloxidase Activity

To assess whether exposure groups (control, low and high-conditioned snail water) differed in phenoloxidase activity, we measured the activity levels of phenoloxidase enzymes through its oxidation of L-dopa on the subsamples of haemolymph (28, 32). Samples were subsampled twice and mixed with 140µL milli-q water and 20µL PBS in in a 96-well microtiter plate. Cold L-dopa (Sigma-Aldrich Canada, Oakville, Canada) solution (4mg/mL in milli-q water) was added to each well and the reaction proceeded at 30°C in a thermocabinet. Four controls per plate (water, PBS, and L-dopa) were created to account for any nonenzymatic oxidation of L-dopa. We measured the absorbance of subsamples at 490nm using a microtitre plate reader (Synergy Neo2, BioTek, Winooski, USA). Absorbance was measured immediately after L-dopa was added (time 0) and at 4hr. These time points were based on preliminary trials, as absorbance increased linearly from 0, 1hr, 2hrs and 4hrs after the addition of L-dopa (Friesen et al. unpublished data). Phenoloxidase activity was calculated as the difference in absorbance between time 0 and each subsequent time point (e.g., absorbance after 4hr – initial absorbance), with the mean difference in the non-haemolymph controls subtracted from the data (32). The phenoloxidase activity is presented in kilo-units.

#### 2.2.2 Statistical Analysis

All statistics were run in R version 4.0.4 (68) and JMP^©^ 16 (69). To test for DDP and determine if snail-conditioned water induced a prophylactic response during a 12hr period, we used Kruskal-Wallis tests to examine if haemocytes or phenoloxidase differed between snails exposed and unexposed to differing densities of snail-conditioned water. Independent variables were treatment (control, low-density snail-conditioned water, and high-density snail-conditioned water) and exposure period (1-2hrs, 4-6hrs, and 8-10hrs). We chose this analysis because after transforming the data in numerous ways (log, ln, exp), the transformed distributions remained non-normal. Normality of the data was verified using the Shapiro–Wilk Test and equal variance was checked with residual plots. *Post-hoc* Steel-Dwass tests were used to determine differences amongst the levels of the main factors.

To determine whether DDP occurred over several weeks, a general linear mixed model was used to assess differences in total haemocyte counts between snails raised in control water or snail-conditioned water. Independent variables were treatment (experiment/control), weeks (time points 1–3), and snail length. The random effect was individual and accounted for repeated measures from the same snail. To assess whether snail-conditioned water affected haemocyte viability, we used general linear mixed modelling with live haemocytes and dead haemocyte numbers as response variables, respectively. Independent variables were treatment (experiment/control), weeks (time points 1–3), and snail length. Haemocyte count was log transformed based on the results of box-cox tests. Model selection began with consideration of a full model including all main effects, covariates, and interactions terms. Components were dropped if they were not significant (*P* > 0.05), and the Akaike information criterion (AIC) value was lower without the component in the model. *Post-hoc* Tukey’s tests were used to determine differences amongst the levels of the main factors.

### 2.3 Experiment 2: Testing the Role of Fatty Acids in Density-Dependent Prophylaxis of Snails

Gas chromatography was used to detect fatty acids in the snail-conditioned and control water from the second trial of experiment 1 (see above, section 2.2). Fatty acids were liquid-liquid extracted from 500mL water samples (one control, one experimental per three weeks) with 125mL methylene chloride. After drying the non-polar fraction in a rotary evaporator, the fatty acids in the samples were transformed to methyl esters following the methods described by Ichihara et al. (2010) (70). Before the methylene chloride extraction, 1µL of a 1mg/mL internal standard of nonadecanoic acid (C19:0) was added to each water sample. We selected C19:0 as an internal standard because in preliminary analyses it was not detected in either type of water. The samples were dried down, re-suspended with 50µL hexane and then analysed by a Varian 450 gas chromatograph equipped with a flame ionization detector. The methylated fatty acids were separated on a DB225MS column (30m × 0.25mm diameter and 0.25m film thickness: Agilent Technologies Canada Inc., Mississauga, Ontario). The temperature program was 70°C for 2 min, then raised to 180°C at 30°C/min, held for 1 min, raised to 200°C at 10 °C/min, held for 2 min, and raised to 220°C at 2°C/min and held for 10 min. The final temperature was raised to 240°C at 20°C/min and held for 15 min. Total run time was 46.67 min, and samples were run with a 20:1 split ratio and a 1.3 mL/min column flow. Hydrogen was used as the carrier gas.

### 2.4 Experiment 3: Characterizing the Oxylipins of Snails Based on Infection Status

We characterized the diversity and amount of oxylipins released by infected and uninfected field-collected *S. elodes* snails. In May-July 2016, *S. elodes* snails were haphazardly collected from the wild and brought back to the laboratory. All field-collected snails were isolated and placed under lights to stimulate the emergence of cercarial parasites to determine if the snails were first intermediate hosts of trematode parasites. Infected snails were separated from uninfected individuals and maintained in the lab for at least two weeks prior to oxylipin extraction. Snails were fed twice weekly an *ad libitum* diet of green leaf lettuce to ensure the same diet.

Live cercariae were obtained from infected first intermediate hosts for identification. Cercariae identified as echinostomes (family Echinostomatidae) based on the presence of collar spines (65) were isolated. Snails that were infected by echinostomes with no tail or fin folds were separated from all other infected snails and maintained in containers with five individuals. As it is difficult to identify echinostome cercariae species based on morphology alone, a subset of parasites was selected for DNA sequencing to verify their identity. We sequenced the DNA at the NADH dehydrogenase 1 (ND1) gene and compared our sequences to ones published on GenBank and our own database to identify the parasites as *Echinoparyphium* sp. lineage 2 (71). Each sample was prepared and analysed as described in detail in Eliuk et al., 2020 (71).

#### 2.4.1 Oxylipin Extraction

We isolated snails into groups of five individuals, based on their infection status, to extract oxylipins from snail-conditioned water. Each group was placed in separate plastic tanks containing 800mL of ASW for 24 hours prior to extraction. We used ASW to minimize biochemical content within the water that did not come from the snails. Snail-conditioned water samples were created by placing each group of snails into a glass jar filled with 50mL of ASW. Shells were wiped cleaned prior to snails being placed in the water. To ensure no contamination, glass jars were cleaned three times with 10% bleach, 70% ethanol, and ASW prior to water collection. After 4 hours, snails were removed from the jars and snail body length was measured. The snail-conditioned water was transferred into sterile tubes and frozen at -20°C until oxylipin extraction.

Snail-conditioned water was thawed just prior to oxylipin extraction. Deuterated internal standard mixtures (Cayman Chemicals, MI) were added to each snail water sample. Samples were then run through 0.2µM filters (Thermo Scientific Nalgene Syringe) to remove any debris (e.g., snail faeces, bacteria, etc.). Solid phase extraction columns (Strata-X, Phenomenex, CA) were created over a vacuum filter apparatus and preconditioned by allowing 100% methanol and pH 3 water drip through. Sample was added to the column and washed with 10% MeOH and pH 3 water. Free oxylipins were finally eluted using 100% methanol. Lipid samples were frozen at -80°C until HPLC/MS/MS analysis (70).

To quantify the oxylipins present in the sample, the lipid samples were dried and resuspended in the initial mobile phase (water/aceto-nitrile/acetic acid, 70/30/0.02, v/v/v). They were then analysed using HPLC/MS/MS (QTRAP 6500; Sciex, ON). Further details of HPLC/MS/MS conditions, including the mass transitions, internal standards, standard curve slopes, retention times, and all oxylipins scanned are described in Aukema et al., 2016, Leng et al., 2017, and Monirujjaman et al., 2017 (72–74).

#### 2.4.2 Statistical Analysis

All data analyses were carried out using JMP^©^ 16 (69). Normality of the data was verified using the Shapiro–Wilk Test and equal variance was checked with residual plots. As data were not normally distributed, we analysed for differences in the amount of oxylipins emitted by infected and uninfected snails using the nonparametric Wilcoxon Ranked Sums. A relationship was considered significant if the *P* value was ≤ 0.05.

### 2.5 Experiment 4: Testing the Role of Oxylipin Signaling Molecules in DDP

**Figure 1:**
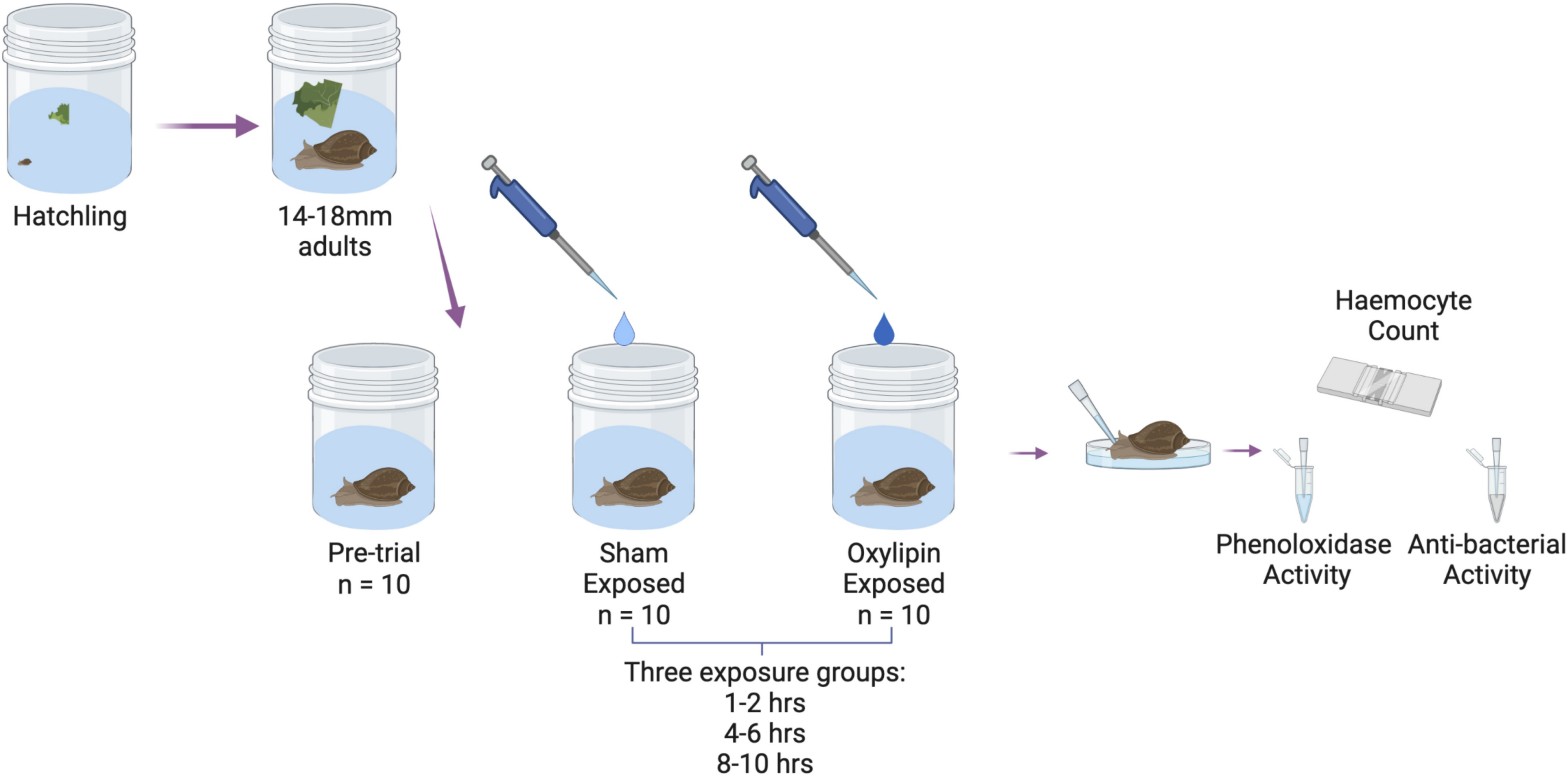
Schematic of experimental design of experiment 4 - testing the role of oxylipin signaling molecules in DDP. Created with BioRender.com.

Snails were raised as described in section 2.2 (trial 1), with all snails derived from only 30 lab colony snails. The snails were allowed to grow to >14mm (mean shell length: 16.06 ± 0.15) to allow for sufficient haemolymph collection from each snail. We monitored reproductive activity, as described in section 2.2. For the experiment, we first randomly chose 10 snails for the pre-trial group, to establish a baseline immune function. For the rest of the experiment, we manipulated the exposure to oxylipins, the type of oxylipin, and exposure time in a 2x4x3 factorial experiment. We sham-exposed or exposed sets of 10 snails, to one of four different oxylipins, 5-hydroxyeicosatetraenoic acid (5-HETE), 15-hydroxyeicosatetraenoic acid (15-HETE), 11-hydroxy-5Z,8Z,12E,14Z,17Z-eicosapentaenoic acid (11-HEPE), or 5-hydroxyeicosapentaenoic Acid (5-HEPE) (**Figure 1**). We then had three different exposure periods, with groups either exposed for 1-2hrs, 4-8hrs, and 8-10hrs (**Figure 1**). Time points were chosen based on the timing of trematode infections from encounter and penetration by the cercarial stage to migrating and encysting near the pericardial sac of the snail (67).

We selected candidate oxylipins for this experiment based on the data obtained from the measurement of oxylipins emitted by infected and uninfected snails that were measured in experiment 3. We chose three oxylipins that were emitted in higher amounts by infected snails (5-HETE, 5-HEPE, and 15-HETE) that are created *via* two different fatty-acid precursors (arachidonic acid and eicosapentaenoic acid), as well as one oxylipin that was only emitted by uninfected snails (11-HEPE, eicosapentaenoic acid). Oxylipin exposure solutions were made by mixing oxylipins (dissolved in ethanol; Cayman Chemical, Ann Arbor, USA) with milli-q water. We used the amount of oxylipins emitted by snail groups measured in experiment 3 to determine the concentration used here. We calculated the concentration of oxylipins for this experiment by accounting for the average oxylipin expressed by a single snail, the average density of this snail species within the environment (11), and the water volume of the jar. Based on this information, we used the following concentrations: 5-HETE – 2.52µg/L, 15-HETE – 3.36µg/L, 5-HEPE – 0.128µg/L, and 11-HEPE – 1.16µg/L. For our sham-exposed controls, we created a solution of ethanol and water at the same concentration as the oxylipins.

The experimental set up was identical to experiment 1, trial 1 (section 2.2). Jars were cleaned, snails added, and then oxylipin and sham-exposure solutions (1mL at desired concentrations) were added to each jar with 69mL of fresh ASW, and snails remained in the water for the entire exposure period. Haemolymph was extracted from each snail and processed as described above for both phenoloxidase assays and haemocyte counts (section 2.2). An additional subsample of 10µL haemolymph was taken for antibacterial assays. Assay samples were mixed with sterilized 100µL phosphate buffered saline (PBS, pH 7.4) and snap frozen with liquid nitrogen. All samples were kept frozen at -80°C until assays could be run.

#### 2.5.1 Antimicrobial Activity

We measured the antibacterial activity of snail haemolymph against wild type *Escherichia coli* K12 cells. Methods were adapted from Seppälä and Jokela (2010) (32). We standardized the *E. coli* cells in 0.85% NaCl to 0.5 MacFarland (1.5 x 10^8^ cells/mL) using a Kit Densichek Plus Instrument (BioMérieux, Marcy-l’Étoile, France). After standardization, cells were then diluted in 1X PBS solution. 200µL of standardized cells were then placed into a 96 well plate following which 50µL of haemolymph sample was mixed in the solution. Each haemolymph sample was subsampled twice. Absorbance of the solution was measured at 450nm using a microplate reader (SpectraMax M2, Molecular Devices, San Jose, United States) immediately after mixing the haemolymph and bacteria with a second read at 30min while incubating at 30°C. In addition, we measured four non-haemolymph controls per plate where the haemolymph was replaced with water to account for any changes in absorbance caused by factors other than antibacterial activity of the haemolymph. Antibacterial activity was then calculated as differences in absorbance between the measurement times (absorbance time 0 – absorbance time 30min, minus internal standard).

#### 2.5.2 Statistical Analysis

To determine whether oxylipin exposure induced snails to produce more haemocytes, a negative binomial generalized linear model was used to assess if differences were due to exposure to oxylipins, type of oxylipin, and period of exposure. Independent variables were exposure (sham control vs. oxylipins: 5-HETE, 15-HETE, 11-HEPE, and 5-HEPE), and exposure length (1-2hrs, 4-6hrs, and 8-10hrs). Due to non-normality and heteroscedasticity of the response variables, generalized linear models of haemocyte count used negative binomial distributions, using *MASS* (75). For phenoloxidase and antibacterial assays, due to the non-normality of the data, Kruskal-Wallis Ranked sums tests were run based on the type of oxylipins used. Posthoc Tukey HSD tests were used to determine differences amongst the levels of the main factors. The model was run on R version 4.0.4 (68) and Kruskal-Wallis tests were run in JMP^©^ 15 (SAS Institute, Inc, 2019).

## 3 Results

### 3.1 Experiment 1: Density-Dependent Prophylaxis in Snails

**Figure 2:**
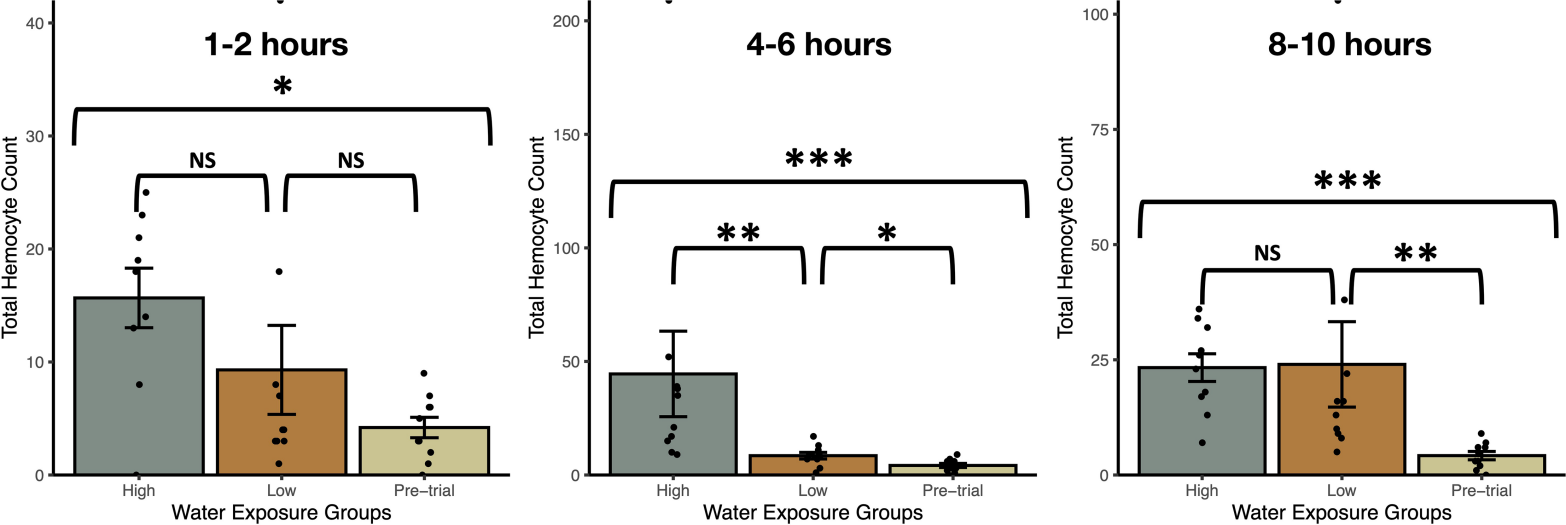
Total haemocyte counts (mean ± SE) from uninfected *Stagnicola elodes* snails exposed to experimental (snail-conditioned water of low and high densities) and unexposed pre-trial snails over three exposure times. N.S, No significative difference; *P < 0.05, **P < 0.01, ***P < 0.001.

**Figure 3:**
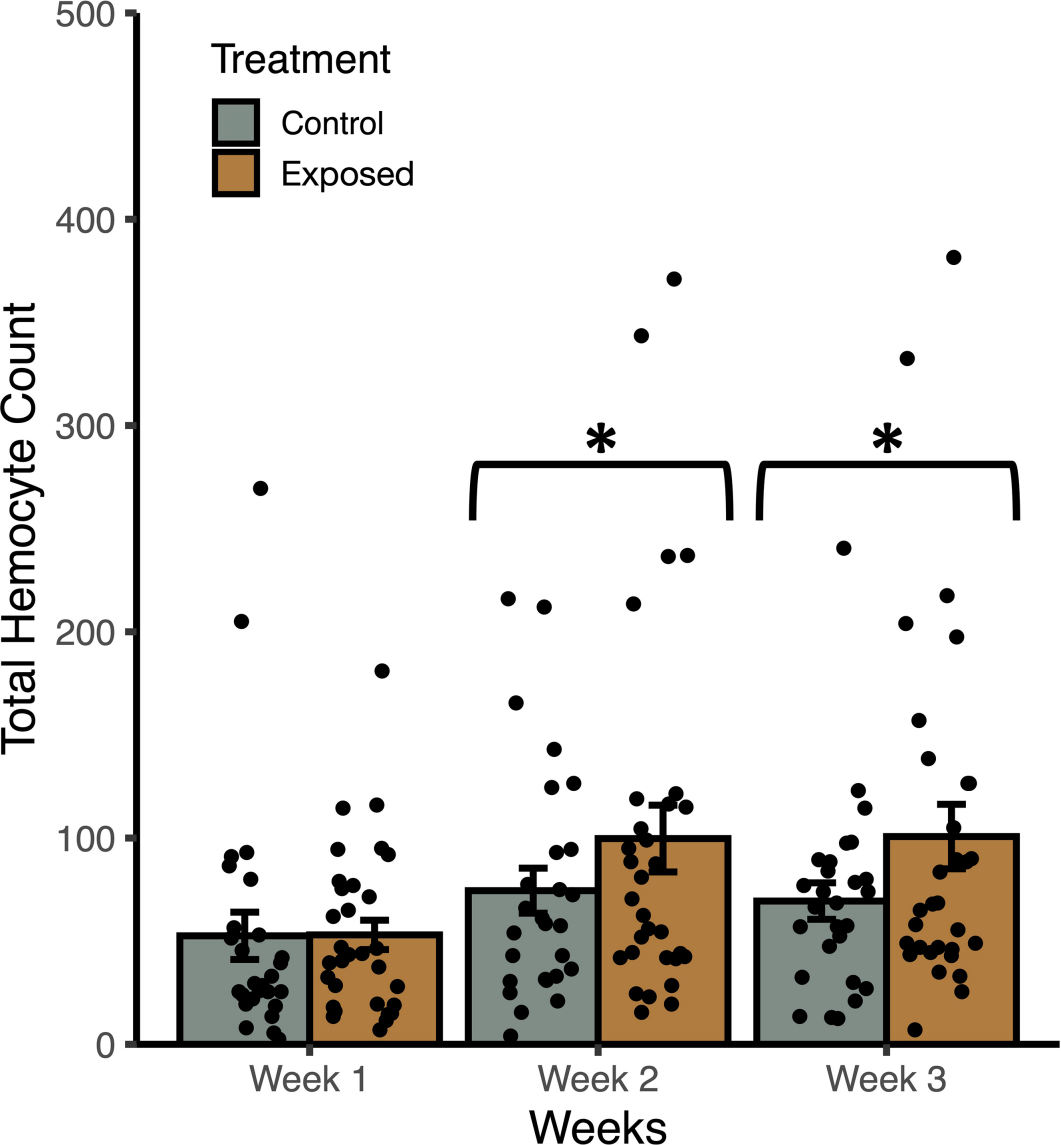
Weekly mean total haemocyte counts (± SE) from uninfected *Stagnicola elodes* snails raised in experimental (snail-conditioned water) and control water. *P < 0.05.

Exposure to snail-conditioned water induced a prophylactic response in snails, both within a 12hr period and over several weeks (**Figures 2**, **3**). In trial 1, haemocyte count differed by treatment over each exposure period (Kruskal-Wallis Ranked sums: 1-2hrs - χ^2^ = 8.2, *P* = 0.0168; 4-6hrs - χ^2^ = 19.2, *P* < 0.0001; 8-10hrs - χ^2^ = 17.6, *P* = 0.0002), with snails exposed to high density water having a higher count than control snails at 1-2hrs (Steel-Dwass: Z = -2.78, *P* = 0.015, **Figure 2**). After 4-6hrs in snail-conditioned water, snails in high density water had the highest haemocyte count and controls had the lowest. Snails exposed to low-density water had an intermediate count between control and high-density exposed snails (control-low, Z = -2.3, *P* = 0.050; low-high, Z = -3.1, *P* = 0.0054; control-high, Z = -3.7, *P* = 0.0006, **Figure 2**). Finally, after 8-10hrs of exposure, there was no difference in haemocytes between snails exposed to high and low-density water (Z = -1.3, *P* = 0.40, **Figure 2**), but snails that were exposed to snail-conditioned water had a higher haemocyte count than control snails (control-low, Z = -3.3, *P* = 0.0028; control-high, Z = -3.6, *P* = 0.0008, **Figure 2**). Exposure to snail-conditioned water did not elicit a phenoloxidase response, with no difference in activity levels between exposure groups or exposure length (Kruskal-Wallis: 1-2hrs exposure: χ^2^ = 1.35, *df* = 2, *P* = 0.51; 4-6hrs: χ^2^ = 5.25, *df* = 2, *P* = 0.073; 8-10hrs: χ^2^ = 3.42, *df* = 2, *P* = 0.18).

In trial 2, snails raised in snail-conditioned water had a higher count of total haemocytes than those raised in control water (F_1, 172_ = 4.56, *P* = 0.037) while accounting for the effects of shell length (F_1, 172 =_ 13.09, *P* = 0.0006) and time (F_2, 171_ = 6.52, *P* = 0.0021) (**Figure 3**). According to AIC values, there was a single best fit model that included treatment groups, snail length, time, and no interactions. The R^2^ of the best model was 0.17 with fixed effects only, and 0.20 with random effects. Similarly, haemocyte viability was greater in snails raised in snail-conditioned because more live haemocytes were found in these snails compared to snails raised in well water (F_1, 172_ = 7.70, *P* = 0.0075) while accounting for the effects of shell length (F_1, 172_ = 14.76, *P* = 0.0003) and time (F_2,_ 171 = 6.94, *P* = 0.0014). The R^2^ of the best model was 0.21 with fixed effects only, and 0.28 with random effects. The number of dead haemocytes did not differ between snails raised in snail-conditioned water and well water (F_1, 172_ = 0.0004, *P* = 0.9839, R^2^ with and without random effects = 0.09).

### 3.2 Experiment 2: Fatty Acids in Snail-Conditioned Water

**Figure 4:**
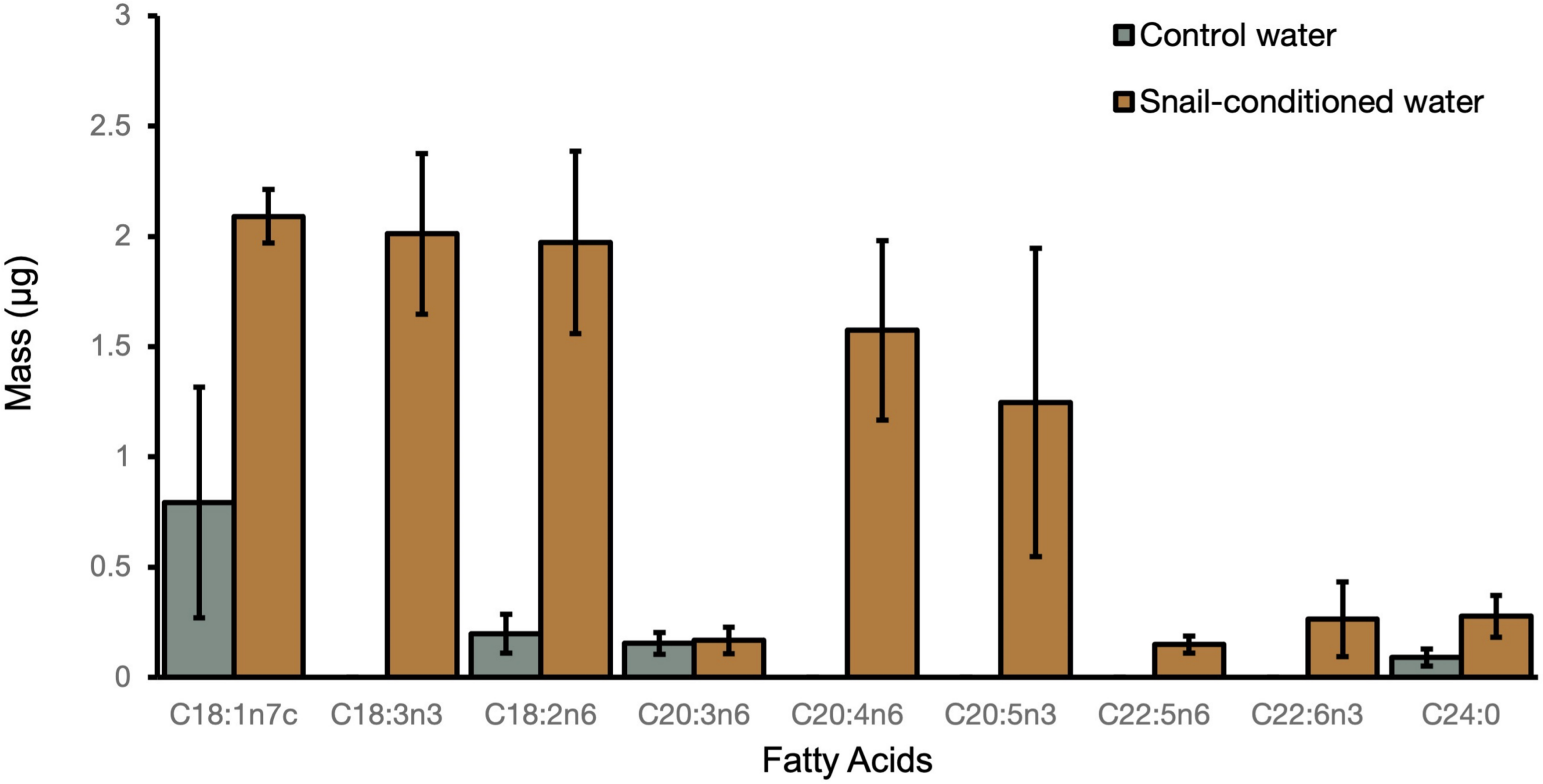
Mean mass of fatty acids ( ± SE) detected in both control and snail-conditioned water over three weeks.

Lipid analysis demonstrated that snails placed in snail-conditioned water were exposed to fatty acid signaling molecules. We detected seven oxylipin precursors including arachidonic acid (ARA, C20:4n-6), eicosapentaenoic acid (EPA, C20:5n-3), α-linolenic acid (ALA, C18:3n-3), docosapentaenoic acid (C22:5n-6) and docosahexaenoic acid (DHA, C22:6n-3) in snail-conditioned but not in control water (**Figure 4**). Linoleic acid (LNA, C18:2n-6) and another oxylipin precursor dihomo-γ-linoleic acid (DGLA, C20:3n-6) were detected in both control and experimental water samples (**Figure 4**).

### 3.3 Experiment 3: Snail Oxylipins Expressed Based on Infection Status

We compared the oxylipins emitted by three groups of infected snails (15 snails) and eight groups of uninfected snails (35 snails). The mean size of infected snails (27.6 ± 0.56) was higher than uninfected snails (18.51 ± 0.43 mm) (Wilcoxon Rank Sums, Z = 2.35, *P* = 0.019).

**Figure 5:**
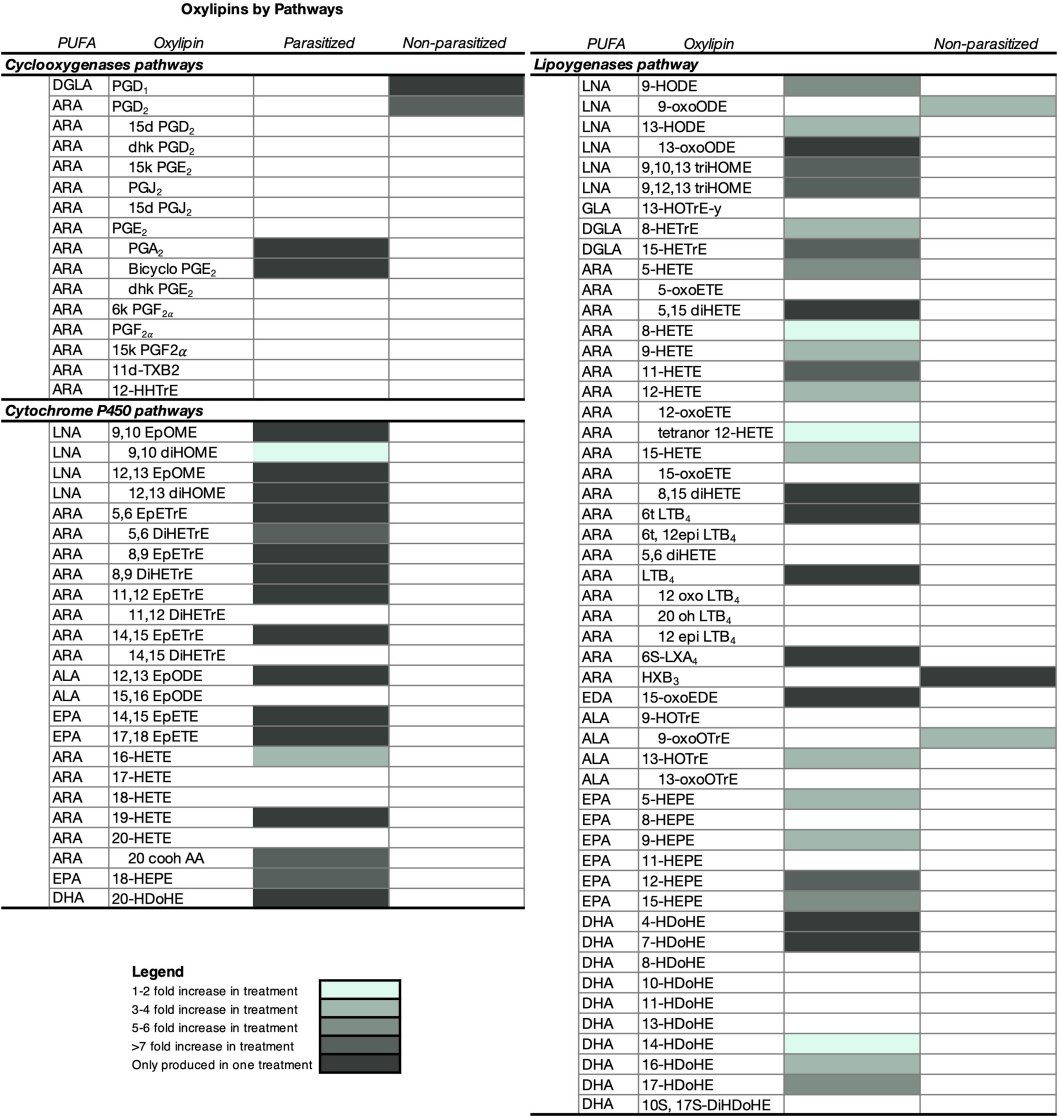
Characterization of oxylipins and relative differences in the amount (mean ± SE) released by parasitized (infected) and non-parasitized (uninfected) field *Stagnicola elodes* snails. Snails in the infected group were parasitized by *Echinoparyphium* sp. lineage 2. All samples were collected from two 250m^2^ ditches along a road bisecting a wetland in Netley-Libau Marsh (50°C 18’48’’N, 96°C 42’29’’W). Oxylipins only detected in one group of snails are also shaded. ARA, arachidonic acid; DGLA, dihomo-g-linolenic acid; LNA, linoleic acid; ALA, a-linolenic acid; ADA, adrenic acid; EPA, eicosapentaenoic acid; DHA, docosahexaenoic acid; DPA, docosapentaenoic acid; GLA, g-linolenic acid; EDA, eicosadienoic acid; d, deoxy; dhk, dihydroketo; DiHDoPE, dihydroxy-docosapentaenoic acid; DiHETE, dihydroxy-eicosatetraenoic acid; DiHETrE, dihydroxy-eicosatrienoic acid; DiHOME, dihydroxy-octadecenoic acid; EpDoPE, epoxy-docosapentaenoic acid; EpODE, epoxy-octadecadienoic acid; EpOME, epoxy-octadecenoic acid; HEPE, hydroxy-eicosapentaenoic acid; HETrE, hydroxy-eicosatrienoic acid; HHTrE, hydroxyheptadecatrienoic acid; HOTrE, hydroxy- octadecatrienoic acid; HX, hepoxilin; LT, leukotriene; t, trans; and Tx, thromboxane.

Out of 157 oxylipins scanned from snail-conditioned water, 95 were present at quantifiable levels (**Figure 5** and **Supplementary Table 1**). According to fatty acid precursors, half (56.8%) of all oxylipins detected were derived from ARA, followed by oxylipins derived from DHA (11.6%), LNA (10.5%), EPA (9.5%), ALA (6.3%), DGLA (3.2%), γ-linolenic acid (C18:3n-6) (1.1%), and eicosadienoic acid (C20:3n-6) (1.1%). But these percentages reflect the proportion of oxylipins derived from each fatty acid, rather than the pathway, which provides a more biologically meaningful parsing of oxylipins. Half (53.7%) of the detected oxylipins were derived *via* the lipoxygenase (LOX) pathway, followed by oxylipins produced from cytochrome P450 (CYP) (25.3%), and cyclooxygenase (COX) (16.8%) pathways. Of the oxylipins produced through the CYP pathway, most (66%) were produced *via* epoxygenase activity.

Overall infected snails emitted 50 oxylipins in higher amounts than the uninfected snails (Wilcoxon Ranked Sums, **Figure 5** and **Supplementary Table 1**, *P* ≤ 0.05), with 24 of these oxylipins only detected in infected snail water (**Figure 5** and **Supplementary Table 1**). A subset of 20 oxylipins were only detected in infected snails, but the amounts did not differ significantly from the amount in uninfected snails (**Supplementary Table 1**). Of the oxylipins expressed in higher amounts by infected snails, oxylipins produced by the LOX pathway dominated (60%), followed by oxylipins derived from CYP (36%) and COX (4%). Interestingly, when looking at oxylipins present only in infected snails, most were derived from the CYP pathway (54%), with 45.8% *via* epoxygenase and 8.3% *via* ω-hydroxylase pathways, compared to 37.5% from the LOX pathway and 8.3% from the COX pathway.

Uninfected snails emitted higher amounts of 5 oxylipins, with 2 of these oxylipins only detected in uninfected snail water with amounts significantly different than infected snails (**Figure 5** and **Supplementary Table 1**). Of the oxylipins detected in higher amounts in uninfected snail water, they were only derived from the COX (40%) and LOX (60%) pathways. A subset of 3 oxylipins were only detected in uninfected snail water, but the amounts did not differ significantly from the amount in infected snails (**Figure 5**).

### 3.4 Experiment 4: Snail Immune Response to Oxylipins

**Figure 6:**
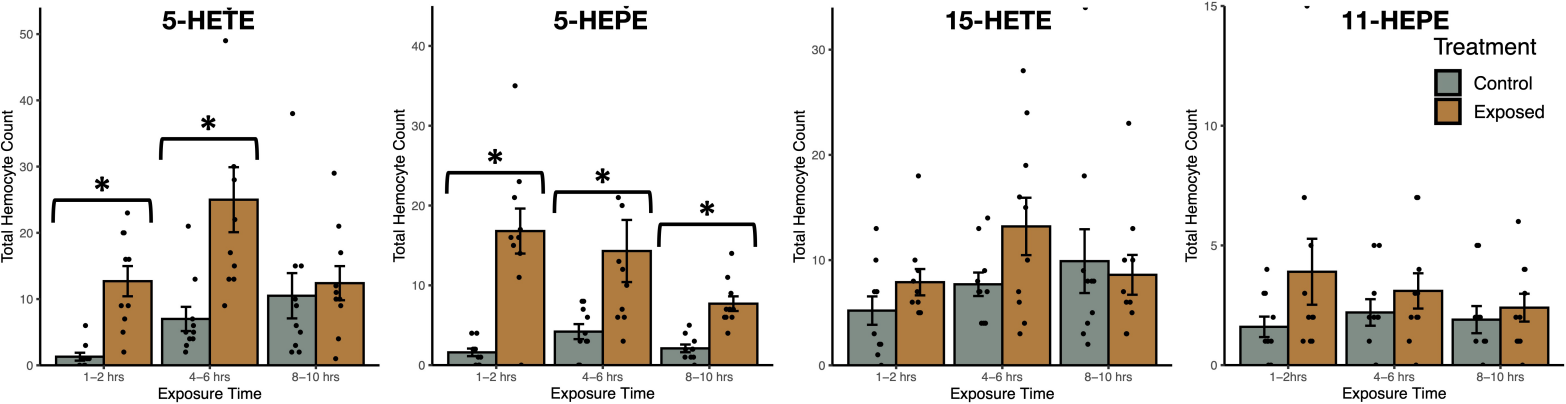
Total haemocyte count (mean ± SE) of *Stagnicola elodes* snails after exposure to 5-Hydroxyeicosatetraenoic acid (5-HETE), 5-Hydroxyeicosapentaenoic Acid (5-HEPE), 15-Hydroxyeicosatetraenoic acid (15-HETE), and 11-hydroxy-5Z,8Z,12E,14Z,17Z-eicosapentaenoic acid (11-HEPE) oxylipins compared to sham-exposed snails. *P < 0.05.

Exposure to oxylipins increased haemocyte count in exposed snails (negative binomial generalized linear model: χ^2^ = 187.8, *df* = 7, *P* < 2.2x10^-16^, **Figure 6**), with exposure time (χ^2^ = 15.3, *df* = 2, *P* = 0.00049) impacting haemocyte count. The interaction between oxylipin exposure and exposure time (χ^2^ = 41.7, *df* = 14, *P* = 0.00014) was significant. Tukey HSD *post-hoc* tests showed that haemocyte count was higher in snails exposed to 5-HETE (z = 5.6, *P* < 0.001) and 5-HEPE (z = 6.0, *P* < 0.001) compared to sham-exposed snails, but there was no difference in haemocyte count in snails exposed to 11-HEPE (z = 2.3, *P* = 0.30) and 15-HETE (z = 1.2, *P* = 0.92). *Post-hoc* tests did not find significant differences between exposure times (*P* > 0.75).

**Figure 7:**
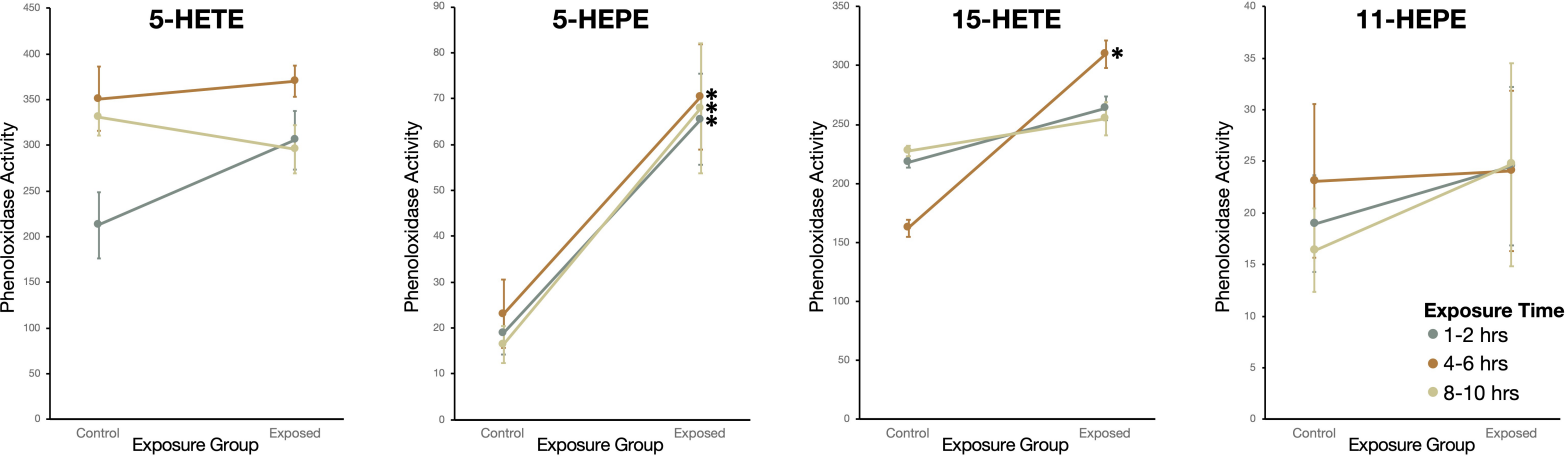
Phenoloxidase (PO) activity (change in absorbance over 2hr measured in kilounits; mean ± SE) of haemolymph in *Stagnicola elodes* snails exposed to 5-Hydroxyeicosatetraenoic acid (5-HETE), 5-Hydroxyeicosapentaenoic Acid (5-HEPE), 15-Hydroxyeicosatetraenoic acid (15-HETE), and 11-hydroxy-5Z,8Z,12E,14Z,17Z-eicosapentaenoic acid (11-HEPE) oxylipins compared to sham-exposed snails. *P < 0.05.

Snails exposed to two oxylipins, 15-HETE and 5-HEPE, had higher phenoloxidase activity than sham-exposed snails (**Figure 7**). Snails exposed to 5-HEPE had higher phenoloxidase activity over every time point (Wilcoxon Rank Sums: 1-2hrs: χ^2^ = 10.6, *df* = 1, *P* = 0.0012; 4-6hrs: χ^2^ = 10.1, *df* =1, *P* = 0.0015; 8-10hrs: χ^2^ = 11.8, *df* = 1, *P* = 0.0006). Exposure to 15-HETE for 4-6hrs induced higher phenoloxidase activity in exposed snails, but there was no difference between exposed and sham-exposed snails after 1-2hrs or 8-10hrs exposure (1-2hrs: χ^2^ = 0.57, *df* = 1, *P* = 0.45; 4-6hrs: χ^2^ = 5.6, *df* =1, *P* = 0.016; 8-10hrs: χ^2^ = 0.46, *df* = 1, *P* = 0.50). Snails exposed to 5-HETE and 11-HEPE did not have more phenoloxidase activity compared to sham-exposed snails (5-HETE - 1-2hrs: χ^2^ = 3.6, *df* = 1, *P* = 0.059; 4-6hrs: χ^2^ = 0.21, *df* =1, *P* = 0.65; 8-10hrs: χ^2^ = 0.76, *df* = 1, *P* = 0.38; 11-HEPE -1-2hrs: χ^2^ = 0.21, *df* = 1, *P* = 0.65; 4-6hrs: χ^2^ = 0.091, *df* =1, *P* = 0.76; 8-10hrs: χ^2^ = 0.0057, *df* = 1, *P* = 0.94).

Exposure to oxylipins only induced increased antibacterial activity in snails after exposure to 5-HETE after a 4-6hr exposure (Wilcoxon Rank Sums, χ^2^ = 5.9, *df* =1, *P* = 0.0155; **Supplementary Figure 1**). However, this increased activity was not seen after 1-2hr exposure (χ^2^ = 0.0057, *df* =1, *P* = 0.94; **Supplementary Figure 1**) or 8-10hr exposure (χ^2^ = 1.12, *df* =1, *P* = 0.29; **Supplementary Figure 1**). Haemolymph from snails exposed to 5-HEPE and 15-HETE did not differ in antibacterial activity compared to sham-controlled snails (5-HEPE: 1-2hrs, χ^2^ = 0.82, *df* =1, *P* = 0.36; 4-6hrs, χ^2^ = 0.36, *df* =1, *P* = 0.059; 8-10hrs, χ^2^ = 1.9, *df* =1, *P* = 0.16; 15-HETE: 1-2hrs, χ^2^ = 0.97, *df* =1, *P* = 0.33; 4-6hrs, χ^2^ = 2.29, *df* =1, *P* = 0.13; 8-10hrs, χ^2^ = 0.14, *df* =1, *P* = 0.71; **Supplementary Figure 1**).

## 4 Discussion

Our study demonstrates that *S. elodes* freshwater snails exhibit DDP and provides evidence that oxylipin signaling molecules are one molecular mechanism for prophylaxis. Our first experiment demonstrated that snails increased their immune defences in response to being exposed to water conditioned with chemical cues from higher densities of snails. *Stagnicola elodes* snails produced more haemocytes in response to perceiving a higher density of snails, suggesting that their immune system is responding to an increased risk of parasite infection. Snails are often aggregated in nature and by doing so can be infected by a variety of different parasites including trematodes, so selection by parasites could have driven this group to evolve this strategy. We further showed that snails raised in snail-conditioned water produced more haemocytes than snails raised in control water, further adding evidence that snails exhibit DDP, and that chemical communication is a mechanism for this behaviour. Our second experiment demonstrated that several of the fatty acids from the snail-conditioned water are biosynthetic precursors of oxylipins that function in the immune defences of invertebrates and vertebrates. In particular, snails produced fatty acids that are precursors of oxylipins, which are signaling molecules that have previously been suggested to play a role in host-parasite interactions (46). In the third experiment that explored the role of chemical communication in DDP, we found vast differences in the oxylipin profile of water conditioned with uninfected and infected snails. This result suggests that differences in oxylipin cocktails may play a role in inducing a DDP response within our snails. Finally, we found that three oxylipins that were higher in infected snails elicited an increase in the immune defences of exposed snails, while the oxylipin present only in uninfected snails elicited no immune responses. This result suggests that particular oxylipins may be the cues that snails are using to detect whether there is an increased risk of parasite infection. Together, the results of these four experiments provide evidence that snails exhibit DDP, and the changes in oxylipins emitted by infected hosts are one of the molecular mechanisms driving this phenomenon.

Our first experiment showed that snails exhibit DDP rapidly in response to increasing density, but also through continued exposure to higher densities of snails. In our first trial, snails exhibited higher counts of haemocytes within 1-2hrs post-exposure that increased further after 4-6hrs. Snails in higher-density water had the highest haemocyte count and snails exposed to low-density snail-conditioned water had a haemocyte count between the unexposed snails and high-density exposure. The differences in haemocyte count between snails in low- and high-density snail-conditioned water suggest that snails are responding accordingly to the threat represented by the difference in snail density, with the higher density representing a higher risk of parasite infection. Yet, after 8-10hrs, snails exposed to low and high density-conditioned water had similar haemocyte counts, which were also lower than both of their responses after 4-6hrs of exposure. It is possible that this difference in response may be related to the chronobiology of parasite infection (76, 77). To maximize the probability of encountering the correct host, many trematode species match the emergence of their short-lived larval parasites, cercariae, with the activity of their next host (76). As such, many trematodes respond to environmental cues of light and heat throughout the day for their emergence, with larval parasite release slowing down or all together stopping during the night. The decreased immune response after 8-10hrs may represent an evolutionary adaptation to reduce energy spent on a costly prophylactic response as the risk of becoming infected declines overnight. Given that snails accumulate trematode infection after repeated exposures over weeks to months and even years, prophylaxis of snails may fluctuate in response to the chronobiology of the parasites as this type of immune response may have been selected for in nature (78). Alternatively, the reduced response after the prolonged exposure may reflect the immune distribution of haemocytes and indicate that they are within other tissues within the snail rather than circulating in the haemolymph. The lack of haemocytes in the haemolymph may indicate that the snails’ immune activity were depleted after a sustained immune defence of several hours. Nevertheless, our second trial shows that snails can recover and sustain a prophylactic response over three weeks while being exposed to snail-conditioned water.

For host density to affect haemocyte production and other measures of immune defences, snails must recognize that they are aggregating. In higher densities, snails could communicate by physical or chemical cues though our experiments focused on chemical communication because a number of studies have suggested that snails communicate through signaling molecules in snail-conditioned water (55, 79–81). We characterized fatty acids from snail water because several of them are the biosynthetic precursors of compounds that function in the immune defences of invertebrates and vertebrates (46, 82). In experiment 2, we demonstrate that snail-conditioned water had five fatty acids that were not detected in control water. Our snail-conditioned water samples were collected from a tank with 60 active, living snails over a period of three weeks demonstrating that grouped snails consistently produced a set of fatty acids though in varying amounts.

The polyunsaturated fatty acids in snail-conditioned water were oxylipin precursors (C20:4n-6, C20:5n-3 and C22:6n-3). Much of the research on oxylipins has focused on vertebrate systems and shows that oxylipins derived from n-6 polyunsaturated fatty acids generally have immunoactive functions and those derived from n-3 polyunsaturated fatty acids have anti-inflammatory properties, although there are some important exceptions (83). Given the increased production of haemocytes of snails in snail-conditioned water as well as snails in higher density-conditioned water, our results suggest that oxylipins may also affect snail immunity. However, in snails, oxylipins are also known to affect egg production and nervous system dynamics (50, 82) though we did not observe any increases in the number of snail eggs in snail-conditioned water (Friesen et al., unpublished data). We also do not know if the origin of these fatty acids is more directly associated with the snail or the parasites as trematodes are also known to produce oxylipins. In schistosome trematode parasites, oxylipins, such as eicosanoids, affect host-finding behaviour of cercariae and may suppress the vertebrate host immune system (56, 84). However, schistosomes and echinostome trematodes differ in many aspects of their biology (i.e., represent different families of trematodes, differ in life cycle complexity, host species used). Further, no study has characterized the oxylipins of echinostome-infected snails despite their ubiquity in freshwater systems (85).

To advance our understanding of the potential role of oxylipins in chemical communication, and as a potential molecular mechanism driving DDP, we needed to characterize more specifically what oxylipins were found within our snail-conditioned water. We also needed to further understand how these chemical cues differed between infected and uninfected snails, as a potential indicator of infection risk to snails within their environment. Snails infected with the trematode parasite *Echinoparyphium* sp. lineage 2 had vastly different oxylipin cocktails than uninfected conspecifics that differed not only in the amount of oxylipins emitted but in the diversity of oxylipins released. We detected 95 different oxylipins emitted from snails, including oxylipins derived from multiple polyunsaturated fatty acids *via* several different pathways (COX, LOX, and CYP), with over half of them either emitted in higher amounts by infected snails or only emitted by infected snails. Despite the fact that the physiological role of these oxylipins is not well understood, other research on many of these oxylipins suggests they may have a role in immune defence and prophylaxis (57, 86, 87). Production of oxylipins by helminth parasites has focused on leukotriene and HETE oxylipins (57, 84, 88, 89). In experiment 3, some of these oxylipins, including 5-HETE and 15-HETE, were emitted in higher amounts in infected snails, suggesting their possible production by echinostome trematodes or echinostome-infected snails.

In experiment 4, responses to 5-HEPE, 5-HETE, and 15-HETE consisted of higher haemocyte counts and phenoloxidase activity, but almost no change in antibacterial activity. This result was predicted given these oxylipins were emitted in higher amounts by infected snails. Conversely, exposure to one oxylipin, 11-HEPE, which was only released by uninfected snails, did not lead to any change in immune defences in exposed snails. The lack of response to 11-HEPE was not unexpected, and further indicates that specific oxylipins stimulate immune defence in snails and may provide a molecular mechanism for DDP. The type of immune defence activated upon exposure varied with oxylipin, suggesting specific oxylipins may play different roles within the snails’ defence system. Exposure to 5-HEPE led to increased haemocyte count and phenoloxidase activity, whereas exposure to 5-HETE only led to increased haemocyte count and exposure to 15-HETE only led to increased phenoloxidase activity. Interestingly, although 5-HETE and 5-HEPE are produced *via* the same enzymes, they are derived from ARA and EPA, respectively, indicating that oxylipins derived from n-6 and n-3 20-carbon fatty acids can have different functions. The activation of the immune defences upon exposure to specific oxylipins allows us start to understand the physiological effects of these oxylipins within snails.

The timing of the immune response was similar among the three oxylipins as well as to the snail-conditioned water alone. In experiment 1 and 4, immune responses started to wane at 8-10hrs post-exposure, bringing up the possibility that the signal had dissipated (e.g., the oxylipin was not being produced or had broken down). If the production of haemocytes is costly, than this change in immune response may suggest exhausted energy reserves, a shift in energy allocation to other life history traits, or a response to the chronobiology of parasite infection (76, 77). However, if the immune response is not costly, further experiments are required to determine how this reduction relates to the immune distribution of haemocytes within different snail tissues.

As DDP in insects is hypothesized to require olfactory, visual, and tactile stimuli acting in concert, it is plausible to suggest that in natural conditions, DDP for snails may be driven by a combination of chemical cues and other signals (2). Within aquatic systems, species obtain important information about their surroundings from chemical cues and individuals can respond to minute changes to concentrations of chemical cues (39, 90, 91). Changes within the oxylipin profiles emitted by infected snails, as we detected here, may be informing conspecifics or other naïve host snails of the risks of infection, eliciting a DDP response that would be beneficial and targeted to the risk. Our results suggest that haemocytes and phenoloxidase activity respond to the risk of eukaryotic infection, and that antimicrobial responses were not part of the DDP response in this set of experiments (23, 24). There is an evolutionary benefit for a snail to respond prophylactically *via* cues from signaling molecules that more specifically indicate whether the risk is from a pathogen or parasite. However, much more research is needed to further understand the generality and strength of alterations to oxylipins in response to infection, how these may vary with host and parasite diversity, and how these changes may impact density-dependent prophylaxis (55). Further, studies on additional invertebrates are needed to understand the evolution of DDP and determine whether similar mechanisms are used across taxa.

Our study expands the number of evolutionary lineages that use DDP in response to the risk of parasitism and suggests that it may be a widely used strategy among invertebrate groups. By further characterizing and testing candidate oxylipins, we have also provided evidence of chemical cues as a mechanism driving density dependent prophylaxis, demonstrating that individual oxylipins may be mediating grouping behaviour and immunity of snails. Yet, this effect has yet to be tested in natural conditions. Some field studies have found negative relationships between snail density and parasite prevalence that were hypothesized to be the result of parasite-induced mortality (95, 96). Low parasite prevalence in high densities suggests that in nature aggregated snails may not be at an increased risk of parasite infection. However, it is important to note that even a single first intermediate host may emit daily 100s-1000s of cercariae that are infective to snail second intermediate hosts. Thus, tests for DDP in nature should quantify infection risk by assessing first intermediate host prevalence and density, and then determine whether co-occurring second intermediate hosts have lower metacercarial infections due to increased investment in their immune systems. In addition to studying this phenomenon in natural populations, future laboratory experiments should explore how oxylipin signals may be detected through the signaling web found within aquatic communities (55). The impacts of these modified chemical cues on the behaviour of other community members, such as other invertebrates and fish, are yet to be fully explored (55). Oxylipins have already been recognized to serve important ecological and physiological functions as mechanisms in tritrophic interactions (97, 98), mediating plant-pathogen and plant-insect interactions (99, 100), and functioning in insect immunity (46). The generality and strength of alterations to oxylipins in response to infection and their immunological and ecological consequences could be assessed across a diversity of aquatic and terrestrial ecosystems. Due to the ubiquity of oxylipins within ecosystems, their importance in immune system function within other species, and their potential role in parasite transmission, and the role of oxylipins in driving DDP in other systems should be explored (42, 46, 50, 55, 57).

## Data Availability Statement

The raw data supporting the conclusions of this article will be made available by the authors, without undue reservation.

## Author Contributions

JD, OF, and C-HL contributed to the conception and design of the study. JD, JS, HA, and AK contributed to methodological design. OF, C-HL, and JD performed the statistical analysis. OF, C-HL, ES, and JD collected the data. OF, C-HL, and ES wrote the first draft of the manuscript. All authors contributed to manuscript revision, read, and approved the submitted version.

## Funding

OF funding was provided by the Weston Family Awards in Northern Research Postdoctoral Fellowship. Financial support for this research was provided by the Natural Sciences and Engineering Research Council of Canada (NSERC) Discovery Grants to JD (RGPIN-2014-05142, RGPIN-2021-02903), HA (RGPIN-06215-2020), and AK (RGPIN-2021-02902), and by the University of Manitoba from the University Research Grant Program (JD) and Interdisciplinary Collaborative grant (JD and HA).

## Conflict of Interest

The authors declare that the research was conducted in the absence of any commercial or financial relationships that could be construed as a potential conflict of interest.

## Publisher’s Note

All claims expressed in this article are solely those of the authors and do not necessarily represent those of their affiliated organizations, or those of the publisher, the editors and the reviewers. Any product that may be evaluated in this article, or claim that may be made by its manufacturer, is not guaranteed or endorsed by the publisher.

## References

[1] Parrish JK Edelstein-Keshet L. Complexity, Pattern, and Evolutionary Trade-Offs in Animal Aggregation. Sci (80- ) (1999) 284:99–101. doi: 10.1126/science.284.5411.99 10102827

[2] Wilson K Cotter SC. Density-Dependent Prophylaxis in Insects. Phenotypic Plast Insects (2009) 44:137–76. doi: 10.1201/b10201-7

[3] Anderson RM May RM. Population Biology of Infectious Diseases: Part I. Nature (1979) 280:361–7. doi: 10.1038/280361a0 460412

[4] Sheldon BC Verhulst S. Ecological Immunology - Costly Parasite Defenses and Trade-Offs in Evolutionary Ecology. Trends Ecol Evol (1996) 11:317–21. doi: 10.1016/0169-5347(96)10039-2 21237861

[5] Kraaijeveld AR Godfray HCJ. Trade-Off Between Parasitoid Resistance and Larval Competitive. Nature (1997) 389:278–80. doi: 10.1038/38483 9305840

[6] Wilson K Reeson AF. Density-Dependent Prophylaxis: Evidence From Lepidoptera-Baculovirus Interactions? Ecol Entomol (1998) 23:100–1. doi: 10.1046/j.1365-2311.1998.00107.x

[7] Wilson K Thomas MB Blanford S Doggett M Simpson SJ Moore SL. Coping With Crowds: Density-Dependent Disease Resistance in Desert Locusts. Proc Natl Acad Sci USA (2002) 99:5471–5. doi: 10.1073/pnas.082461999 PMC122793 11960003

[8] Barnes AI Siva-Jothy MT. Density-Dependent Prophylaxis in the Mealworm Beetle *Tenebrio Molitor* L. (Coleoptera: Tenebrionidae): Cuticular Melanization is an Indicator of Investment in Immunity. Proc R Soc B Biol Sci (2000) 267:177–82. doi: 10.1098/rspb.2000.0984 PMC1690519 10687824

[9] Silva FWS Serrão JE Elliot SL. Density-Dependent Prophylaxis in Primary Anti-Parasite Barriers in the Velvetbean Caterpillar. Ecol Entomol (2016) 41:451–8. doi: 10.1111/een.12315

[10] Mills SC. Density-Dependent Prophylaxis in the Coral-Eating Crown-of-Thorns Sea Star. Acanthaster Planci Coral Reefs (2012) 31:603–12. doi: 10.1007/s00338-012-0883-2

[11] McKillop WB. Distribution of Aquatic Gastropods Across the Ordovician Dolomite - Precambrian Granite Contact in Southeastern Manitoba, Canada. Can J Zool (1985) 63:278–88. doi: 10.1139/z85-043

[12] Lewis DB Magnuson JJ. Landscape Spatial Patterns in Freshwater Snail Assemblages Across Northern Highland Catchments. Freshw Biol (2000) 43:409–20. doi: 10.1046/j.1365-2427.2000.00514.x

[13] Simpson AW Thomas JD Townsend CR. Social Behavior in the Freshwater Pulmonate Snail *Biomphalaria Glabrata* (Say). Behav Biol (1973) 9:731–40. doi: 10.1016/S0091-6773(73)80133-6 4764254

[14] Lodge DM. Macrophyte-Gastropod Associations: Observations and Experiments on Macrophyte Choice by Gastropods. Freshw Biol (1985) 15:695–708. doi: 10.1111/j.1365-2427.1985.tb00243.x

[15] Lodge DM Brown KM Klosiewski SP Stein RA Covich AP Leathers BK. Distribution of Freshwater Snails: Spatial Scale and the Relative Importance of Physicochemical and Biotic Factors. Am Malacol Bull (1987) 5:73–84.

[16] Lorencová E Horsák M. Environmental Drivers of Mollusc Assemblage Diversity in a System of Lowland Lentic Habitats. Hydrobiologia (2019) 9:49–64. doi: 10.1007/s10750-019-3940-9

[17] Littlewood DTJ Bray RA. Interrelationships of the Platyhelminthes. London; New York: Taylor & Francis (2000).

[18] Poulin R. Global Warming and Temperature-Mediated Increases in Cercarial Emergence in Trematode Parasites. Parasitology (2006) 132:143–51. doi: 10.1017/S0031182005008693 16393363

[19] Preston DL Orlofske SA Lambden JP Johnson PTJ. Biomass and Productivity of Trematode Parasites in Pond Ecosystems. J Anim Ecol (2013) 82:509–17. doi: 10.1111/1365-2656.12030 23488451

[20] Cort WW Brackett S Olivier L. Lymnaeid Snails as Second Intermediate Hosts of the Strigeid Trematode, *Cotylurus Flabelliformis* (Faust, 1917). J Parasitol (1944) 30:309. doi: 10.2307/3272581

[21] Herrmann KK Sorensen RE. Seasonal Dynamics of Two Mortality-Related Trematodes Using an Introduced Snail. J Parasitol (2009) 95:823–8. doi: 10.1645/GE-1922.1 20049988

[22] Keeler SP Huffman JE. Echinostomes in the Second Intermediate Host. In: Fried B Toledo R, editors. The Biology of Echinostomes. New York, NY: Springer. (2009).

[23] van der Knaap WPW Loker ES. Immune Mechanisms in Trematode-Snail Interactions. Parasitol Today (1990) 6:175–82. doi: 10.1016/0169-4758(90)90349-9 15463334

[24] Pila EA Li H Hambrook JR Wu X Hanington PC. Schistosomiasis From a Snail’s Perspective: Advances in Snail Immunity. Trends Parasitol (2017) 33:845–57. doi: 10.1016/j.pt.2017.07.006 28803793

[25] Sminia T Borghart-Reinders E van de Linde AW. Encapsulation of Foreign Materials Experimentally Introduced Into the Freshwater Snail *Lymnaea Stagnalis* - An Electron Microscopic and Autoradiographic Study. Cell Tissue Res (1974) 153:307–26. doi: 10.1007/BF00229161 4617627

[26] van der Knaap WPW Adema CM Sminia T. Invertebrate Blood Cells: Morphological and Functional Aspects of the Haemocytes in the Pond Snail *Lymnaea Stagnalis* . Comp Haematol Int (1993) 3:20–6. doi: 10.1007/BF00394923

[27] Kryukova NA Yurlova NI Rastyagenko NM Antonova EV Glupov VV. The Influence of *Plagiorchis Mutationis* Larval Infection on the Cellular Immune Response of the Snail Host *Lymnaea Stagnalis* . J Parasitol (2014) 100:284–7. doi: 10.1645/13-214.1 24428684

[28] Cerenius L Söderhäll K. The Prophenoloxidase-Activating System in Invertebrates. Immunol Rev (2004) 198:116–26. doi: 10.1111/j.0105-2896.2004.00116.x 15199959

[29] Le Clec’h W Anderson TJC Chevalier FD. Characterization of Hemolymph Phenoloxidase Activity in Two *Biomphalaria* Snail Species and Impact of *Schistosoma Mansoni* Infection. Parasites Vectors (2016) 9:1–11. doi: 10.1186/s13071-016-1319-6 26797101 PMC4722754

[30] Durrant HJ Ratcliffe NA Hipkin CR Aspan A Soderhall K. Purification of the Pro-Phenol Oxidase Enzyme From Haemocytes of the Cockroach *Blaberus Discoidalis* . Biochem J (1993) 289:87–91. doi: 10.1042/bj2890087 8424776 PMC1132134

[31] Cerenius L Lee BL Söderhäll K. The proPO-System: Pros and Cons for its Role in Invertebrate Immunity. Trends Immunol (2008) 29:263–71. doi: 10.1016/j.it.2008.02.009 18457993

[32] Seppälä O Jokela J. Maintenance of Genetic Variation in Immune Defense of a Freshwater Snail: Role of Environmental Heterogeneity. Evol (N Y) (2010) 64:2397–407. doi: 10.1111/j.1558-5646.2010.00995.x 20298461

[33] Imler J Bulet P. Antimicrobial Peptides in Drosophila: Structures,Activities and Gene Regulation. Chem Immunol Allergy (2005) 86:1–21. doi: 10.1159/000086648 15976485

[34] Langand J Jourdane J Coustau C Delay B Morand S. Cost of Resistance, Expressed as a Delayed Maturity, Detected in the Host-Parasite System *Biomphalaria Glabrata/Echinostoma Caproni* . Heredit (Edinb) (1998) 80:320–5. doi: 10.1046/j.1365-2540.1998.00291.x

[35] Rigby MC Jokela J. Predator Avoidance and Immune Defence: Costs and Trade-Offs in Snails. Proc R Soc B Biol Sci (2000) 267:171–6. doi: 10.1098/rspb.2000.0983 PMC1690515 10687823

[36] Seppälä O Leicht K. Activation of the Immune Defence of the Freshwater Snail *Lymnaea Stagnalis* by Different Immune Elicitors. J Exp Biol (2013) 216:2902–7. doi: 10.1242/jeb.084947 23842628

[37] Brönmark C Hansson L-A. Chemical Communication in Aquatic Systems: An Introduction. Oikos (2000) 88:103–9. doi: 10.1034/j.1600-0706.2000.880112.x

[38] Van Poecke RMP Dicke M. Indirect Defence of Plants Against Herbivores: Using *Arabidopsis Thaliana* as a Model Plant. Plant Biol (2004) 6:387–401. doi: 10.1055/s-2004-820887 15248121

[39] Vos M Vet LEM Wäckers FL Middelburg JJ van der Putten WH Mooij WM. Infochemicals Structure Marine, Terrestrial and Freshwater Food Webs: Implications for Ecological Informatics. Ecol Inform (2006) 1:23–32. doi: 10.1016/j.ecoinf.2005.06.001

[40] Kats LB Dill LM. The Scent of Death : Chemosensory Assessment of Predation Risk by Prey Animals. Ecoscience (1998) 5:361–94. doi: 10.1080/11956860.1998.11682468

[41] Burks RL Lodge DM. Cued in: Advances and Opportunities in Freshwater Chemical Ecology. J Chem Ecol (2002) 28:1901–17. doi: 10.1023/A:1020785525081 12474890

[42] Fink P. Ecological Functions of Volatile Organic Compounds in Aquatic Systems. Mar Freshw Behav Physiol (2007) 40:155–68. doi: 10.1080/10236240701602218

[43] Karowe DN Pearce TA Spaller WR. Chemical Communication in Freshwater Snails: Behavioral Responses of *Physa Parkeri* to Mucous Trails of *P. Parkeri* (Gastropoda:Pulmonata) and *Campeloma Decisum* (Gastropoda:Prosobranchia). Malacol Rev (1993) 26:9–14.

[44] Marcopoulos AA Fried B. Intraspecific and Interspecific Chemoattraction in *Biomphalaria Glabrata* and *Helisoma Trivolvis* (Gastropoda: Planorbidae). J Chem Ecol (1994) 20:2645–51. doi: 10.1007/BF02036198 24241838

[45] Pohnert G. Phospholipase A_2_ Activity Triggers the Wound-Activated Chemical Defense in the Diatom. Thalassiosira Rotula Plant Physiol (2002) 129:103–11. doi: 10.1104/pp.010974 PMC155875 12011342

[46] Stanley DW. Eicosanoids in Invertebrate Immunity. In: Eicosanoids in Invertebrate Signal Transduction Systems. Princeton, New Jersey: Princeton University Press. (2016). p. 109–51.

[47] Wilson RA. An Investigation Into the Mucus Produced by *Lymnaea Truncatula*, the Snail Host of *Fasciola Hepatica* . Comp Biochem Physiol (1968) 24:629–33. doi: 10.1016/0010-406X(68)91016-5 5651298

[48] Chaffee LA Fried B Sherma J. Neutral Lipids in Snail-Conditioned Water From *Biomphalaria Glabrata* (Gastropoda: Planorbidae). J Chem Ecol (1996) 22:231–5. doi: 10.1007/BF02055095 24227406

[49] Rivas F Fried B Sherma J. Neutral Lipids in Snail Conditioned Water From Two Strains of *Helisoma Trivolvis* (Gastropoda: Planorbidae). Microchem J (1997) 56:114–21. doi: 10.1006/mchj.1996.1441

[50] Stanley-Samuelson DW. The Biological Significance of Prostaglandines and Related Eicosanoids in Invertebrates. Am Zool (1994) 34:589–98. doi: 10.1093/icb/34.6.589

[51] Wall R Ross RP Fitzgerald GF Stanton C. Fatty Acids From Fish: The Anti-Inflammatory Potential of Long-Chain Omega-3 Fatty Acids. Nutr Rev (2010) 68:280–9. doi: 10.1111/j.1753-4887.2010.00287.x 20500789

[52] Stanley-Samuelson DW Jensen E Nickerson KW Tiebel K Ogg CL Howard RW. Insect Immune Response to Bacterial Infection is Mediated by Eicosanoids. Proc Natl Acad Sci USA (1991) 88:1064–8. doi: 10.1073/pnas.88.3.1064 PMC50955 1899480

[53] Morishima I Yamano Y Inoue K Matsuo N. Eicosanoids Mediate Induction of Immune Genes in the Fat Body of the Silkworm, *Bombyx Mori* . FEBS Lett (1997) 419:83–6. doi: 10.1016/S0014-5793(97)01418-X 9426224

[54] Caldwell GS. The Influence of Bioactive Oxylipins From Marine Diatoms on Invertebrate Reproduction and Development. Mar Drugs (2009) 7:367–400. doi: 10.3390/md7030367 19841721 PMC2763107

[55] Friesen OC Detwiler JT. Parasite-Modified Chemical Communication: Implications for Aquatic Community Dynamics. Front Ecol Evol (2021) 9:634754:634754. doi: 10.3389/fevo.2021.634754

[56] Allan F Rollinson D Smith JE Dunn AM. Host Choice and Penetration by *Schistosoma Haematobium* Miracidia. J Helminthol (2009) 83:33–8. doi: 10.1017/S0022149X08073628 18922204

[57] Noverr MC Erb-Downward JR Huffnagle GB. Production of Eicosanoids and Other Oxylipins by Pathogenic Eukaryotic Microbes. Clin Microbiol Rev (2003) 16:517–33. doi: 10.1128/CMR.16.3.517-533.2003 PMC164223 12857780

[58] Chaisson KE Hallem EA. Chemosensory Behaviours of Parasites. Trends Parasitol (2012) 28:427–36. doi: 10.1016/j.pt.2012.07.004.Chemosensory PMC5663455 22921895

[59] Gordy MA Kish L Tarrabain M Hanington PC. A Comprehensive Survey of Larval Digenean Trematodes and Their Snail Hosts in Central Alberta, Canada. Parasitol Res (2016) 115:3867–80. doi: 10.1007/s00436-016-5152-9 27245072

[60] Detwiler JT Bos DH Minchella DJ. Revealing the Secret Lives of Cryptic Species: Examining the Phylogenetic Relationships of Echinostome Parasites in North America. Mol Phylogenet Evol (2010) 55:611–20. doi: 10.1016/j.ympev.2010.01.004 20064622

[61] Detwiler JT Zajac AM Minchella DJ Belden LK. Revealing Cryptic Parasite Diversity in a Definitive Host: Echinostomes in Muskrats. J Parasitol (2012) 98:1148–55. doi: 10.1645/GE-3117.1 22694483

[62] Kanev I Fried B Dimitrov V Radev V. Redescription of *Echinostoma Trivolvis* (Cort, 1914) (Trematoda: Echinostomatidae) With a Discussion on its Identity. Syst Parasitol (1995) 32:61–70. doi: 10.1007/BF00009468 7707213

[63] Burch JB. North American Freshwater Snails.—365. Hamburg S, editor. Michigan (81-215 Nachdruck aus Walkerana: Society for Experimental and Descriptive Malacology (1980) p. 217–365.

[64] Clarke AH. The Freshwater Molluscs of Canada. Ottawa, Canada: National Museum of Natural Sciences, National Museums of Canada (1981).

[65] Schell SC. Trematodes of North America, North of Mexico. Moscow, Idaho: University Press of Idaho (1985).

[66] Ulmer MJ. Notes on Rearing of Snails in the Laboratory. In: MacInnis AJ Voge M, editors. Experiments and Techniques in Parasitology. San Francisco: W.H. Freeman and Company. (1970). p. 143–4.

[67] Anderson JW Fried B. Experimental Infection of *Physa Heterostropha*, *Helisoma Trivolvis*, and *Biomphalaria Glabrata* (Gastropoda) With *Echinostoma Revolutum* (Trematoda) Cercariae. J Parasitol (1987) 73:49–54. doi: 10.2307/3282342 3572665

[68] R Core Development Team. R: A Language and Environment for Statistical Computing. (2011). Vienna Austria: R Foundation for Statistical Computing.

[69] SAS Institute Inc. JMP (R) Version 16. (2021). North Carolina: SAS Institute Inc.

[70] Ichihara K Fukubayashi Y. Preparation of Fatty Acid Methyl Esters for Gas-Liquid Chromatography. J Lipid Res (2010) 51:635–40. doi: 10.1194/jlr.D001065 PMC2817593 19759389

[71] Eliuk LK Brown S Wyeth RC Detwiler JT. Parasite-Modified Behaviour in Non-Trophic Transmission: Trematode Parasitism Increases the Attraction Between Snail Intermediate Hosts. Can J Zool (2020) 98:417–24. doi: 10.1139/cjz-2019-0251

[72] Aukema HM Winter T Ravandi A Dalvi S Miller DW Hatch GM. Generation of Bioactive Oxylipins From Exogenously Added Arachidonic, Eicosapentaenoic and Docosahexaenoic Acid in Primary Human Brain Microvessel Endothelial Cells. Lipids (2016) 51:591–9. doi: 10.1007/s11745-015-4074-0 26439837

[73] Leng S Winter T Aukema HM. Dietary LA and Sex Effects on Oxylipin Profiles in Rat Kidney, Liver, and Serum Differ From Their Effects on PUFAs. J Lipid Res (2017) 58:1702–12. doi: 10.1194/jlr.M078097 PMC5538291 28667077

[74] Monirujjaman M Devassy JG Yamaguchi T Sidhu N Kugita M Gabbs M. Distinct Oxylipin Alterations in Diverse Models of Cystic Kidney Diseases. Biochim Biophys Acta - Mol Cell Biol Lipids (2017) 1862:1562–74. doi: 10.1016/j.bbalip.2017.08.005 28826940

[75] Venables WN Ripley BD. Modern Applied Statistics with S, Fourth edition. New York: Springer (2002). ISBN 0-387-95457-0

[76] Théron A. Chronobiology of Trematode Cercarial Emergence: From Data Recovery to Epidemiological, Ecological and Evolutionary Implications. Elsevier Ltd (2015) 88:123–64. doi: 10.1016/bs.apar.2015.02.003 25911367

[77] Toledo R Muioz-antoli C Esteban JG. Production and Chronobiology of Emergence of the Cercariae of *Euparyphium Albuferensls* (Trematoda: Echinostomatidae). J Parasitol (1999) 85:263–7. doi: 10.2307/3285630 10219306

[78] Curtis LA. The Probability of a Marine Gastropod Being Infected by a Trematode. J Parasitol (1996) 82:830–3. doi: 10.2307/3283899 8885896

[79] Townsend CR. Mucus Trail Following by the Snail *Biomphalaria Glabrata* (Say). Anim Behav (1974) 22:170–7. doi: 10.1016/S0003-3472(74)80066-7

[80] Erlandsson J Kostylev V. Trail Following, Speed and Fractal Dimension of Movement in a Marine Prosobranch, *Littorina Littorea*, During a Mating and a Non-Mating Season. Mar Biol (1995) 122:87–94. doi: 10.1007/BF00349281

[81] Clifford KT Gross L Johnson K Martin KJ Shaheen N Harrington MA. Slime-Trail Tracking in the Predatory Snail, *Euglandina Rosea* . Behav Neurosci (2003) 117:1086–95. doi: 10.1037/0735-7044.117.5.1086 14570557

[82] Stanley-Samuelson DW. Comparative Eicosanoid Physiology in Invertebrate Animals. Am J Physiol - Regul Integr Comp Physiol (1991) 260:R849–53. doi: 10.1152/ajpregu.1991.260.5.r849 2035696

[83] Gabbs M Leng S Devassy JG Aukema HM. Advances in Our Understanding of Oxylipins. Am Soc Nutr (2015) 6:513–40. doi: 10.3945/an.114.007732.PUFAs PMC4561827 26374175

[84] Fusco AC Salafsky B Delbrook K. *Schistosoma Mansoni*: Production of Cercarial Eicosanoids as Correlates of Penetration and Transformation. J Parasitol (1986) 72:397–404. doi: 10.2307/3281679 3746561

[85] Bolek MG Detwiler JT Stigge HA. Selected Wildlife Trematodes. In: Toledo R Fried B, editors. Digenetic Trematodes. Cham: Springer International Publishing. (2019) p. 321–55. doi: 10.1007/978-3-030-18616-6_11

[86] Weinberger F Lion U Delage L Kloareg B Potin P Beltrán J. Up-Regulation of Lipoxygenase, Phospholipase, and Oxylipin-Production in the Induced Chemical Defense of the Red Alga *Gracilaria Chilensis* Against Epiphytes. J Chem Ecol (2011) 37:677–86. doi: 10.1007/s10886-011-9981-9 21671082

[87] Rempt M Weinberger F Grosser K Pohnert G. Conserved and Species-Specific Oxylipin Pathways in the Wound-Activated Chemical Defense of the Noninvasive Red Alga *Gracilaria Chilensis* and the Invasive *Gracilaria Vermiculophylla* . Beilstein J Org Chem (2012) 8:283–9. doi: 10.3762/bjoc.8.30 PMC3302091 22423296

[88] Fusco AC Salafsky B Kevin MB. *Schistosoma Mansoni*: Eicosanoid Production by Cercariae. Exp Parasitol (1985) 59:44–50. doi: 10.1016/0014-4894(85)90055-4 3917929

[89] Baset HA O’Neill GP Ford-Hutchinson AW. Characterization of Arachidonic-Acid-Metabolizing Enzymes in Adult *Schistisoma Mansoni* . Mol Biochem Parasitol (1995) 73:31–41. doi: 10.1016/0166-6851(95)00085-F 8577345

[90] Brönmark C Hansson L-A. Chemical communication in aquatic systems: An introduction. Oikos (2000) 86:103–9. doi: 10.1034/j.1600-0706.2000.880112.x

[91] Dicke M Sabelis MW. Infochemical Terminology: Based on Cost-Benefit Analysis Rather Than Origin of Compounds? Funct Ecol (1988) 2:131. doi: 10.2307/2389687

[92] Cotter SC Hails RS Cory JS Wilson K. Density-Dependent Prophylaxis and Condition-Dependent Immune Function in Lepidopteran Larvae: A Multivariate Approach. J Anim Ecol (2004) 73:283–93. doi: 10.1111/j.0021-8790.2004.00806.x

[93] Ruiz-González MX Moret Y Brown MJF. Rapid Induction of Immune Prophylaxis in Adult Social Insects. Biol Lett (2009) 5(6):781–3. doi: 10.1098/rsbl.2009.0505 PMC2828006 19656864

[94] Kong H Cheng Y Luo L Sappington TW Jiang X Zhang L. Density-Dependent Prophylaxis in Crowded Beet Webworm, Loxostege Sticticalis (Lepidoptera: Pyralidae) Larvae to a Parasitoid and a Fungal Pathogen. Int J Pest Manag (2013) 59:174–9. doi: 10.1080/09670874.2013.807957

[95] Lafferty KD. Effects of Parasitic Castration on Growth, Reproduction and Population Dynamics of the Marine Snail *Cerithidea Californica* . Mar Ecol Prog Ser (1993) 96:229–37. doi: 10.3354/meps096229

[96] Puurtinen M Knott KE Suonpää S Van Ooik T Kaitala V. Genetic Variability and Drift Load in Populations of an Aquatic Snail. Evol (N Y) (2004) 58:749–56. doi: 10.1111/j.0014-3820.2004.tb00408.x 15154551

[97] Kergunteuil A Humair L Maire AL Moreno-Aguilar MF Godschalx A Catalán P. Tritrophic Interactions Follow Phylogenetic Escalation and Climatic Adaptation. Sci Rep (2020) 10:1–10. doi: 10.1038/s41598-020-59068-2 32034273 PMC7005781

[98] Weissburg MJ Beauvais J. The Smell of Success: The Amount of Prey Consumed by Predators Determines the Strength and Range of Cascading Non-Consumptive Effects. PeerJ (2015) 3:e1426. doi: 10.7717/peerj.1426 26618090 PMC4655096

[99] Cellini A Buriani G Rocchi L Rondelli E Savioli S Rodriguez Estrada MT. Biological Relevance of Volatile Organic Compounds Emitted During the Pathogenic Interactions Between Apple Plants and Erwinia Amylovora. Mol Plant Pathol (2018) 19:158–68. doi: 10.1111/mpp.12509 PMC6637988 27862864

[100] Arimura GI Kost C Boland W. Herbivore-Induced, Indirect Plant Defences. Biochim Biophys Acta - Mol Cell Biol Lipids (2005) 1734:91–111. doi: 10.1016/j.bbalip.2005.03.001 15904867

