# Supplementary material for: Density-Dependent Prophylaxis in Freshwater Snails Driven by Oxylipin Chemical Cues
Source: Front Immunol. 2022 Jan 31;13:826500. doi: 10.3389/fimmu.2022.826500 (PMC8841777; doi:10.3389/fimmu.2022.826500)
Supplement: Supplementary file 1 [file DataSheet_1.pdf]

Supplementary Material

1 Supplementary Figures and Tables

Supplementary Table 1. Differences in amount of oxylipins (mean ± SE) released by infected and uninfected field *Stagnicola elodes* snails. Snails in the infected group were parasitized by *Echinoparyphium* sp. lineage 2. All samples were collected from two 250m^2 ditches along a road bisecting a wetland in Netley-Libau Marsh (50°18'48''N, 96°42'29''W). Acronyms: ARA, arachidonic acid; DGLA, dihomo-γ-linolenic acid; LNA, linoleic acid; ALA, α-linolenic acid; ADA, adrenic acid; EPA, eicosapentaenoic acid; DHA, docosahexaenoic acid; GLA, γ-linolenic acid; EDA, eicosadienoic acid; d, deoxy; dhk, dihydroketo; DiHETE, dihydroxy-eicosatetraenoic acid; DiHETrE, dihydroxy-eicosatrienoic acid; DiHOME, dihydroxy-octadecenoic acid; DPA, docosapentaenoic acid; EpODE, epoxy-octadecadienoic acid; EpOME, epoxy-octadecenoic acid; HEPE, hydroxy-eicosapentaenoic acid; HETE, hydroxy-eicosatetraenoic acid; HETrE, hydroxy-eicosatrienoic acid; HHTrE, hydroxyheptadecatrienoic acid; HODE, hydroxy-octadecadienoic acid; HOTrE, hydroxy-octadecatrienoic acid; HX, hepoxilin; LT, leukotriene; oxoODE, oxo-octadecadienoic acid; oxoETE, oxo-eicosatetraenoic acid; t, trans; TriHOME, trihydroxyoctadecenoic acid; and Tx, thromboxane.

PUFA	Pathways	Oxylipin	Parasitized (n=3) Mean ± SE (ng)	Nonparasitized (n=8) Mean ± SE (ng)	Z	*P*
DGLA	COX	PGD_1	0	0.142 ± 0.0433	-2.37	**0.018**
ARA	COX	PGD_2	0.0419 ± 0.0419	0.568 ± 0.105	-2.15	**0.032**
ARA	COX	15d PGD_2	0.0150 ± 0.0150	0	1.43	0.15
ARA	COX	dhk PGD_2	0.308 ± 0.0801	0.289 ± 0.0545	0	1
ARA	COX	15k PGE_2	0.0927 ± 0.0260	0.134 ± 0.0301	-0.51	0.61
ARA	COX	PGJ_2	0.0239 ± 0.0239	0	1.43	0.15
ARA	COX	15d PGJ_2	0	0.0484 ± 0.0241	-1.04	0.3
ARA	COX	PGE_2	0.262 ± 0.209	0.377 ± 0.0994	-0.92	0.358
ARA	COX	PGA_2	0.0517 ± 0.0267	0	2.27	**0.023**
ARA	COX	Bicyclo PGE_2	0.192 ± 0.0695	0	2.99	**0.003**
ARA	COX	dhk PGE_2	0.0148 ± 0.0148	0	1.43	0.15
ARA	COX	6k $PGF_{1\alpha}$	0.0112 ± 0.0112	0	1.49	0.15
ARA	COX	$PGF_{2\alpha}$	0.171 ± 0.0951	0.144 ± 0.0192	0	1
ARA	COX	15k $PGF_{2\alpha}$	0.011 ± 0.011	0	1.43	0.15
ARA	COX	11d-TXB_2	0.0308 ± 0.0308	0	1.43	0.15
ARA	COX	12-HHTrE	0.0341 ± 0.0641	0	1.43	0.15

LNA	CYP	9,10 EpOME	0.104 ± 0.0274	0	2.99	**0.003**
LNA	CYP	9,10 DiHOME	0.0529 ± 0.00748	0.0238 ± 0.00242	2.35	**0.019**
LNA	CYP	12,13 EpOME	0.0607 ± 0.0154	0	2.99	**0.003**
LNA	CYP	12,13 DiHOME	0.0740 ± 0.0379	0	2.27	**0.023**
ARA	CYP	5,6 EpETrE	0.0917 ± 0.0359	0	2.99	**0.003**
ARA	CYP	5,6 DiHETrE	0.187 ± 0.0951	0.0016 ± 0.0016	2.72	**0.007**
ARA	CYP	8,9 EpETrE	0.120 ± 0.0160	0	2.99	**0.003**
ARA	CYP	8,9 DiHETrE	0.0119 ± 0.00599	0	2.27	**0.023**
ARA	CYP	11,12 EpETrE	0.0706 ± 0.0262	0	2.99	**0.003**
ARA	CYP	11,12 DiHETrE	0.0206 ± 0.00704	0.0138 ± 0.0017	0.919	0.36
ARA	CYP	14,15 EpETrE	0.211 ± 0.0602	0	2.99	**0.003**
ARA	CYP	14,15 DiHETrE	0.0353 ± 0.00778	0.0385 ± 0.00724	-0.1	0.92
ALA	CYP	12,13 EpODE	0.128 ± 0.0213	0	2.99	**0.003**
ALA	CYP	15,16 EpODE	0	0.00676 ± 0.00676	-0.41	0.68
EPA	CYP	14,15 EpETE	0.0610 ± 0.0307	0	2.27	**0.023**
EPA	CYP	17,18 EpETE	0.149 ± 0.0582	0	2.99	**0.003**
ARA	CYP	16-HETE	0.341 ± 0.0695	0.122 ± 0.0233	2.35	**0.019**
ARA	CYP	17-HETE	0.0077 ± 0.0077	0	1.43	0.15
ARA	CYP	18-HETE	0.0665 ± 0.0176	0.0327 ± 0.0102	1.53	0.13
ARA	CYP	19-HETE	0.662 ± 0.173	0	2.99	**0.003**
ARA	CYP	20-HETE	0.0239 ± 0.0239	0	1.43	0.15
ARA	CYP	20 cooh AA	5.39 ± 2.50	0.793 ± 0.153	2.35	**0.019**
EPA	CYP	18-HEPE	1.97 ± 0.433	0.241 ± 0.0372	2.34	**0.019**
DHA	CYP	20-HDoHE	0.0791 ± 0.0126	0	2.98	**0.003**
LNA	LOX	9-HODE	4.76 ± 1.02	0.829 ± 0.123	2.35	**0.019**
LNA	LOX	9-oxoODE	1.35 ± 0.365	3.45 ± 0.596	-2.14	**0.032**
LNA	LOX	13-HODE	3.45 ± 0.649	0.820 ± 0.0984	2.35	**0.019**
LNA	LOX	13-oxoODE	5.28 ± 0.143	0	2.99	**0.003**
LNA	LOX	9,10,13 triHOME	7.16 ± 0.956	1.08 ± 0.310	2.35	**0.019**
LNA	LOX	9,12,13 triHOME	5.99 ± 2.06	0.470 ± 0.079	2.35	**0.019**
GLA	LOX	13-HOTrE-y	0.409 ± 0.0616	0.267 ± 0.0549	1.74	0.082
DGLA	LOX	8-HETrE	0.170 ± 0.0146	0.044 ± 0.0095	2.35	**0.019**
DGLA	LOX	15-HETrE	1.49 ± 0.111	0.00935 ± 0.0268	2.35	**0.019**
ARA	LOX	5-HETE	2.83 ± 0.411	0.528 ± 0.058	2.35	**0.019**

ARA	LOX	5-oxoETE	0.522 ± 0.143	0.688 ± 0.380	0.71	0.48
ARA	LOX	5,15 DiHETE	0.206 ± 0.0136	0	2.99	**0.003**
ARA	LOX	8-HETE	3.56 ± 0.663	1.589 ± 0.260	2.35	**0.019**
ARA	LOX	9-HETE	3.46 ± 1.06	0.952 ± 0.182	2.35	**0.019**
ARA	LOX	11-HETE	6.41 ± 1.56	0.159 ± 0.014	2.35	**0.019**
ARA	LOX	12-HETE	3.92 ± 0.692	0.980 ± 0.215	2.35	**0.019**
ARA	LOX	12-oxoETE	0.305 ± 0.0608	6.45 ± 4.03	0.54	0.59
ARA	LOX	tetranor 12-HETE	0.124 ± 0.0267	0.0542 ± 0.0115	2.14	**0.032**
ARA	LOX	15-HETE	3.77 ± 0.188	0.905 ± 0.122	2.34	**0.019**
ARA	LOX	15-oxoETE	1.53 ± 0.268	2.28 ± 0.576	-0.51	0.61
ARA	LOX	8,15 DiHETE	1.08 ± 0.208	0	2.99	**0.003**
ARA	LOX	6t LTB_4	0.242 ± 0.176	0	2.27	**0.023**
ARA	LOX	6t, 12epi LTB_4	0.231 ± 0.231	0	1.43	0.15
ARA	LOX	5,6 DiHETE	0.0074 ± 0.0074	0	1.43	0.15
ARA	LOX	LTB_4	0.0528 ± 0.0103	0	2.99	**0.003**
ARA	LOX	12 oxo LTB_4	0.0208 ± 0.0208	0	1.43	0.15
ARA	LOX	20 oh LTB_4	0.0122 ± 0.0122	0	1.43	0.15
ARA	LOX	12 epi LTB_4	0.0200 ± 0.0200	0	1.43	0.15
ARA	LOX	6S-LXA_4	0.670 ± 0.121	0	2.99	**0.003**
EDA	LOX	15-oxoEDE	0.501 ± 0.118	0	2.99	**0.003**
ALA	LOX	9-HOTrE	2.62 ± 0.707	2.024 ± 0.902	1.74	0.083
ALA	LOX	9-oxoOTrE	0.890 ± 0.227	3.32 ± 0.684	-2.14	**0.032**
ALA	LOX	13-HOTrE	7.81 ± 2.21	2.08 ± 0.368	2.35	**0.019**
ALA	LOX	13-oxoOTrE	3.68 ± 3.68	0	1.43	0.15
EPA	LOX	5-HEPE	1.31 ± 0.106	0.448 ± 0.156	2.14	**0.032**
EPA	LOX	8-HEPE	0.383 ± 0.114	0.534 ± 0.144	-0.31	0.76
EPA	LOX	9-HEPE	1.57 ± 0.293	0.485 ± 0.171	2.16	**0.031**
EPA	LOX	11-HEPE	0	0.0949 ± 0.0202	-1.72	0.086
EPA	LOX	12-HEPE	2.92 ± 0.529	0.396 ± 0.0704	2.35	**0.019**
EPA	LOX	15-HEPE	3.99 ± 0.739	0.686 ± 0.0603	2.35	**0.019**
DHA	LOX	4-HDoHE	0.178 ± 0.038	0	2.99	**0.003**
DHA	LOX	7-HDoHE	0.0545 ± 0.0278	0	2.27	**0.023**
DHA	LOX	8-HDoHE	0.163 ± 0.0474	0.170 ± 0.0628	0	1
DHA	LOX	10-HDoHE	0.0370 ± 0.0075	0.0283 ± 0.0111	0.721	0.47

DHA	LOX	11-HDoHE	0.0675 ± 0.00783	0.181 ± 0.150	0.73	0.47
DHA	LOX	13-HDoHE	0.178 ± 0.0239	0.0335 ± 0.0296	1.89	0.059
DHA	LOX	14-HDoHE	0.190 ± 0.0415	0.0794 ± 0.0140	2.14	**0.03**
DHA	LOX	16-HDoHE	0.102 ± 0.00806	0.0328 ± 0.00595	2.35	**0.019**
DHA	LOX	17-HDoHE	0.511 ± 0.0506	0.106 ± 0.0342	2.37	**0.018**
DHA	LOX	10S, 17S-DiHDoHE	0.00557 ± 0.00557	0	1.43	0.15
ARA	non-enz	2,3-dinor 8-iso $PGF_{2\alpha}$	0.025 ± 0.025	0	1.43	0.15
ARA	non-enz	5-iso $PGF_{2\alpha VI}$	0.0089 ± 0.00089	0	1.43	0.15
ARA	non-enz	8-iso $PGF_{2\alpha III}$	0.00269 ± 0.00269	0	1.43	0.15
ARA	Synthetic	15d PGA_2	0.226 ± 0.226	0	1.43	0.15

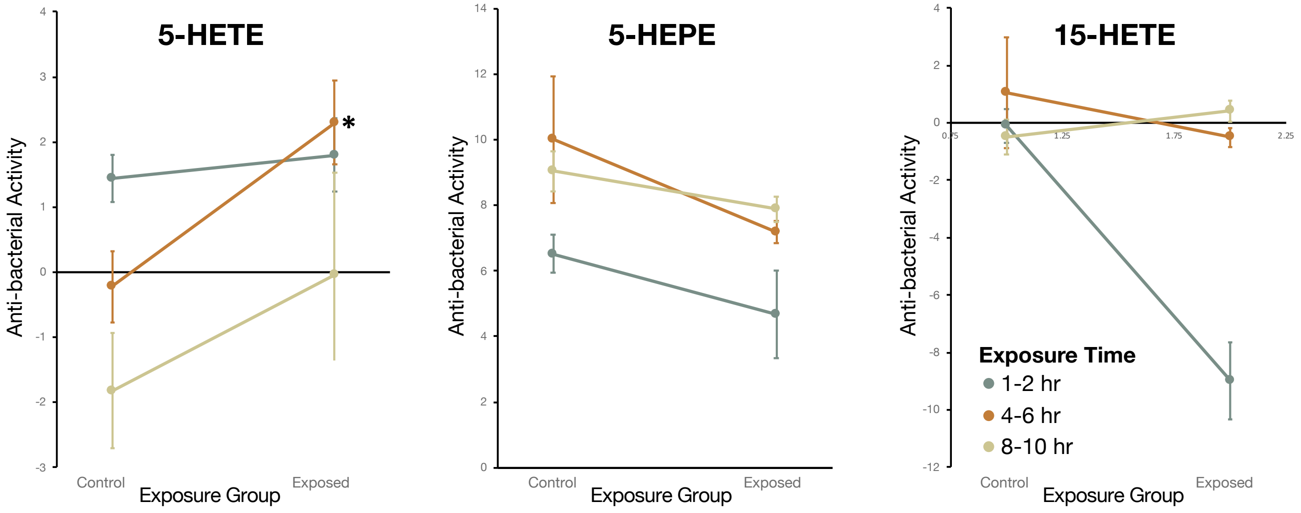

Supplementary Figure 1 Anti-bacterial (AB) activity (change in absorbance over 30 minutes measured in kilounits; mean ± SE) of haemolymph in *Stagnicola elodes* snails exposed to 5-Hydroxyeicosatetraenoic acid (5-HETE), 5-Hydroxyeicosapentaenoic Acid (5-HEPE), and 15-Hydroxyeicosatetraenoic acid (15-HETE) oxylipins compared to sham-exposed snails. *$p<0.05$.
